# TNFAIP8 regulates autophagy, cell steatosis, and promotes hepatocellular carcinoma cell proliferation

**DOI:** 10.1038/s41419-020-2369-4

**Published:** 2020-03-09

**Authors:** Suryakant Niture, Maxwell A. Gyamfi, Minghui Lin, Uchechukwu Chimeh, Xialan Dong, Weifan Zheng, John Moore, Deepak Kumar

**Affiliations:** 1 Julius L. Chambers Biomedical Biotechnology Research Institute, North Carolina Central University Durham, Durham, NC 27707 USA; 2 Ningxia Medical University, Ningxia Hui Autonomous Region, Yinchuan, 750004 China; 3 Department of Pharmaceutical Sciences, Bio-manufacturing Research Institute and Technology Enterprise (BRITE), North Carolina Central University Durham, Durham, NC 27707 USA; 4 Department of Pharmaceutical Sciences, North Carolina Central University Durham, Durham, NC 27707 USA

**Keywords:** Oncogenes, Macroautophagy

## Abstract

Tumor necrosis factor-α-induced protein 8 (TNFAIP8) expression has been linked to tumor progression in various cancer types, but the detailed mechanisms of TNFAIP8 are not fully elucidated. Here we define the role of TNFAIP8 in early events associated with development of hepatocellular carcinoma (HCC). Increased TNFAIP8 levels in HCC cells enhanced cell survival by blocking apoptosis, rendering HCC cells more resistant to the anticancer drugs, sorafenib and regorafenib. TNFAIP8 also induced autophagy and steatosis in liver cancer cells. Consistent with these observations, TNFAIP8 blocked AKT/mTOR signaling and showed direct interaction with ATG3-ATG7 proteins. TNFAIP8 also exhibited binding with fatty acids and modulated expression of lipid/fatty-acid metabolizing enzymes. Chronic feeding of mice with alcohol increased hepatic levels of TNFAIP8, autophagy, and steatosis but not in high-fat-fed obese mice. Similarly, higher TNFAIP8 expression was associated with steatotic livers of human patients with a history of alcohol use but not in steatotic patients with no history of alcohol use. Our data indicate a novel role of TNFAIP8 in modulation of drug resistance, autophagy, and hepatic steatosis, all key early events in HCC progression.

## Introduction

Tumor necrosis factor-α-induced protein 8 (TNFAIP8) is a member of the TNFAIP8/TIPE family, which also includes TIPE1, TIPE2 and TIPE3^1–4^. In cancer cells, expression of TNFAIP8 is associated with cell survival and drug resistance^2,5^. TNFAIP8 contains a death effector domain, which negatively regulates cell apoptosis^2^ and is involved in numerous human diseases associated with inflammation, infection, and immunity^6^. By inhibiting cell apoptosis, TNFAIP8 promotes cancer cell proliferation and drug resistance^7–12^. In human cancer, depletion of TNFAIP8 has been shown to increase the expression of genes associated with anti-proliferation and apoptosis (*IL-24, FAT3*, *LPHN2*, *EPHA3*), fatty-acid oxidation (*ACADL*), and decrease the expression of several oncogenes such as *NFAT5*, *MALAT1*, *MET*, *FOXA1*, *KRAS*, *S100P*, *OSTF1*^5^. In HCC cells, expression of TNFAIP8 induces cell proliferation, migration, invasion, and xenograft tumor growth^13^. TNFAIP8 modulates the Hippo pathway by inhibition of YAP phosphorylation and by the interaction with LATS1 protein. TNFAIP8-LATS1 interaction increases nuclear localization and stabilization of YAP protein, resulting in increased cell proliferation. Knockdown of LATS1 or YAP by siRNA blocks the effects of TNFAIP8 on cell proliferation, suggesting that TNFAIP8 promotes HCC progression through LATS1-YAP signaling pathway^13^.

Macroautophagy (here referred to as “autophagy”) is a cellular response to metabolic stress which plays a role in oncogenesis^14^. We recently demonstrated that TNFAIP8 induces autophagy, drug resistance, and cell proliferation in prostate and breast cancer cells^12^. Similarly, in an earlier study, it was demonstrated that a loss-of-function of TNFAIP8 homolog, CG4091/Sigmar, led to decreased autophagic flux in *Drosophila*^15^. Sigmar interacts with several cytoskeletal proteins and the kinase Misshapen, which activate autophagy, both directly and indirectly^15^. On the other hand, induction of TNFAIP8 by insulin inhibits autophagy by the formation of TNFAIP8-phosphatidylethanolamine (PE)-Gαi3 ternary complex in mouse hepatoma Hepa-1-6 cells^16^. Under oxidative stress conditions, TNFAIP8 interacts with F-Box and WD repeat domain containing 5 (FBXW5) and stabilizes tubular sclerosis complex 2 (TSC2), a negative regulator of mTOR, and thus enhances autophagy in dopaminergic neurons^17^.

Here, we demonstrate that TNFα mediated induction of TNFAIP8 or overexpression of TNFAIP8 inhibits the AKT/mTOR pathway and mediates autophagy induction. Importantly, TNFAIP8 interacts with the ATG3-ATG7 autophagosome complex proteins and promotes LC3 lipidation, a crucial process for functional autophagy. We found that TNFAIP8 not only promoted liver cancer cell survival/drug resistance but also increased cell/hepatic steatosis in alcoholic fatty liver diseases (AFLD) suggesting that TNFAIP8–autophagy may contribute to early liver cancer progression by modulation of hepatic steatosis in mice and in human patients.

## Materials and methods

### Mice and reagents

All animal procedures were carried out in accordance with the NIH Guidelines for the Care and Use of Laboratory Animals and approved by the NCCU Institutional Animal Care and Use Committee (IACUC). The NCCU-IACUC Protocol No. is MG-02-26-2010. Six-week-old male C57BL/6J mice were purchased from The Jackson laboratory (Bar Harbor, ME, USA). TNFAIP8-Myc-DDK-tagged ORF cDNA plasmid (Cat # RC202729) was obtained from Origene (Rockville, MD), TNFAIP8-his tagged protein (Cat # 14559-H07E) from Sino Biological (Wayne, PA), TNFAIP8 siRNA (Cat # L-020589), control siRNA (Cat # D-001830) from Dharmacon (Lafayette, CO), Oil Red O stain (Cat # O0625 from Sigma (St. Louis, MO), CYTO-ID® Autophagy Detection Kit (Cat # ENZ-51031-0050) from Enzo Life Science (Farmingdale, NY), G418 (Geneticin) (Cat # 11811-031) from Gibco (Gaithersburg, MD), MTT reagent (Cat # 102227) from MP Biomedical (Solon, OH), CellRox green reagents (Cat # C10444) from Molecular Probes, regorafenib (Cat # R8024) from LC Laboratories, sorafenib (Cat # S1040) from Selleckchem, HCC-TMA slide (Cat # BC03116) from US Biomax (Rockville, MD), TMA-Alcohol Steatosis (Cat # TMA.AS 1810055) and TMA No Alcohol Steatohepatitis (Cat # TMA. NASH 1810177) from Sekisui Xeno Tech (Kansas City, KS) and human liver normal and tumor tissue lysates from Protein Biotechnologies (Ramona, CA). The following antibodies were obtained from Cell Signaling Technology (Danvers, MA): anti-LC3β I/II (Cat # 4108S), anti-4E-BP1 (Cat # 9452S), anti-cPARP (Cat # 9541S), anti-Caspase-3 (Cat # 9662S), anti-AKT (Cat # 9272S), anti-pS473-AKT (Cat # 3787S), anti-mTOR (Cat # 2983S), anti-pS2448-mTOR (Cat # 2971S), anti-fatty-acid synthase (FASN) (Cat #, 3180S), anti-SCD1 (Cat # 2794S), anti-ACC (Cat # 3662S), anti-Myc tag (Cat # 2278S), anti-ATG7 (Cat # 8558S), anti-GAPDH (Cat # 5174S), and anti-β-actin (Cat # 4970S). We also purchased anti-PPRA-γ (Cat # sc-7273), anti-SREBP1 (Cat # sc-13551), and anti-Beclin-1 (Cat # sc-4834) from Santa Cruz Biotechnology (Dallas, TX); anti-ATG3 (Cat # A3231) from Sigma (St. Louis, MO); anti-L-FABP (Cat # ab7366) from Abcam; anti-TNFAIP8 (Cat # 15790-1-AP) antibody from Proteintech (Rosemont, IL).

### Cell culture

Liver cancer cell lines, HepG2, SK-Hep1, PLC/PRF5, and Hep3B were obtained from Georgetown University (GU) Lombardi Comprehensive Cancer Center (LCCC) cell culture repository. Liver cancer cells were grown in DMEM medium (Invitrogen) supplemented with 5% Fetal Bovine Serum (FBS, Access Biologicals, Vista, CA) and 50 U/ml penicillin/streptomycin (Cellgro) and incubated at 37 °C in a cell culture incubator supplied with 5% CO_2_. We also generated a stable HepG2 cell line which expressed TNFAIP8-Myc-tagged protein. HepG2 cells were transfected with empty vector (pcDNA3.1) or TNFAIP8-Myc-tagged plasmid for 24 h. Cells were trypsinized, re-plated, and treated with Geneticin (G418) (600 µg/ml) for 2 weeks. Antibiotic*-*containing medium was replaced every 3–4 days; cell colonies were selected, expanded, and maintained in the presence of G418. The TNFAIP8-Myc-tagged protein expression was confirmed by immunoblotting. Cell lines used in this study were tested for mycoplasma contamination and were authenticated by STR sequencing. All cells lines were grown for at least 24 h and after 70–80% of confluence, cells were used for the experiments.

### Western blot

HCC cells (1 × 10^5^) were grown in 6-well plates for 24 h. Cells were transfected with indicated plasmids or treated with anticancer drugs or autophagy inhibitors. Cells were washed with PBS and lysed with cell lysis buffer (Cell Signaling, Danvers, MA) containing protease inhibitor mixture (Roche, Indianapolis, IN) and PMSF (0.1 mM). Protein concentrations were determined using the Bio-Rad protein assay reagent (Bio-Rad, Hercules, CA). The proteins (40–60 µg) were separated by NuPAGE 4–12% Bis-Tris-SDS gel (Invitrogen) and transferred to a PVDF membrane (Thermo Scientific, Rockford, IL). Membranes were blocked in 1X blocking buffer (Sigma-Aldrich, St. Louis, MO) for 1 h and incubated with indicated primary antibodies for overnight at 4 °C. All antibodies were used as per the manufacturer’s suggestions. After washing the membranes three times with Tris-buffered saline with 0.1% Tween 20 (TBST), the membranes were incubated in the appropriate secondary antibody (1:10,000 dilution) (Jackson ImmunoResearch, PA) for 1 h at room temperature, and immunoreactive bands were visualized using ECL chemiluminescence detection reagents (Signagen Laboratories, Rockville, MD). The blots were developed appropriately with band detection within linear range, and intensities were quantified using ImageJ software (National Institutes of Health).

### Tissue microarray (TMA) and immunohistochemical analysis (IHC)

Tissue microarray slides containing liver tumor tissues (*n* = 40) and their corresponding adjacent normal tissues (*n* = 13) were obtained from US Biomax (Rockville, MD). Tissue microarray slides containing liver steatosis tissue with history of alcohol use (*n* = 19), without alcohol use (*n* = 5), and comparative normal liver tissues (*n* = 5), as well as slides containing steatohepatitis (NASH) tissue with alcohol use (*n* = 5), without alcohol use (*n* = 12), and normal liver tissues (*n* = 5) were obtained from Sekisui Xeno Tech LLC (Kansas City, KS). Paraffin-embedded four-micron tissue sections were stained with TNFAIP8 antibody (1:50 dilution) by incubation at 4 °C overnight. Vector Labs Elite ABC KIT used for detection of TNFAIP8 staining, with 3,3′-diaminobenzidine as the chromogen and hematoxylin as the counterstain (Innovex Biosciences). Slides were assessed by pathologists (University of North Carolina, Chapel Hill, NC) who were blinded to any associated experimental results or outcomes. TMA slides were digitalized in Aperio ScanScope XT (Leica) using ×20 objective (0.468 μm/pixel resolution) at the UNC Translational Pathology Core Lab. TMA slides were saved and segmented for analysis in Aperio eSlide Manager using TMA Lab software. TNFAIP8 staining was analyzed in the individual cores using Aperio Color Deconvolution Algorithm v9, and TNFAIP8 expression scores were obtained.

### RNA isolation, cDNA synthesis, and RT/qPCR

HepG2, SK-Hep1, PLC/PRF5, and Hep3B cells were plated in 6-well plates at a density of 1 × 10^5^ cells/well for 24 h. Cells were harvested, washed with PBS, and total RNA was isolated using TRIZOL Reagent (Invitrogen, Carlsbad, CA). The expression of TNFAIP8 different isoforms was analyzed by using isoforms specific primers (Supplementary Table 1) followed by RT/PCR as described previously^12^. In another experiment, HepG2, SK-Hep1, and Hep3B cells were transfected with empty vector or TNAIP8-Myc plasmid for 24 h, and total RNA was isolated using TRIZOL Reagent. RNA (1 µg) was reverse transcribed using a High Capacity cDNA Reverse Transcription kit (Applied Biosystems, Carlsbad, CA). cDNA was mixed with Power SYBR Green PCR master mix (Applied Biosystems) with both forward and reverse specific primers of the TNFAIP8, IL-6 genes, or lipid/fatty-acid metabolizing enzymes (Supplementary Table 1). GAPDH was amplified as an internal control. The PCR mixtures were run on a QuantStudio-3 PCR System (Applied Biosystems) using relative quantitation according to the manufacturer’s protocols.

### Plasmid and siRNA transfections

Human tumor necrosis factor-alpha-induced protein 8, transcript variant 1 (TNFAIP8)-Myc-DDK-tagged ORF cDNA plasmid was obtained from Origene (Rockville, MD). The plasmid encodes 198 amino acids of TNFAIP8 protein, with Myc tag (EQKLISEEDL), and a FLAG-tag (DYKDDDDK). For transfection, HepG2, SK-Hep1, and Hep3B liver cancer cells were plated in 6-well plates at a density of 1 × 10^5^ cells/well for 24 h. Cells were transfected with 0.5–1 μg of the pcDNA3.1 empty vector (EV) or TNFAIP8-Myc-tagged plasmid DNA using Lipofectamine LTX-plus transfection reagent (Invitrogen) according to the manufacturer’s instructions. After 24 h of transfection, cells were harvested, and expression of the TNFAIP8-Myc-tagged protein was examined by immunoblotting. For siRNA transfection, we used control siRNA and human TNFAIP8 siRNA purchased from Dharmacon (Lafayette, CO). HepG2, SK-Hep1, and Hep3B cells were transfected with 100 nM control or TNFAIP8 siRNA using Lipofectamine RNAiMAX reagent (Invitrogen) according to the manufacturer’s instructions. Following 24 h of transfection, cells were harvested, and TNFAIP8 knockdown was confirmed by immunoblotting the lysates with the anti-TNFAIP8 antibody.

### MTT assay

MTT cell survival assay was performed in 96-well plates using MTT (3-(4,5-dimethylthiazol-2-yl)-2,5-diphenyl tetrazolium bromide) reagent. HCC cells were transfected with EV or TNFAIP8-Myc plasmid DNA for 24 h alone or treated with various concentration of sorafenib or regorafenib for 48 h. Similarly, EV or TNFAIP8-stable-expressing HepG2 cells or TNFAIP8-knockdown HepG2 and SK-Hep1 cells were treated with sorafenib or regorafenib for 48 h as indicated. After 48 h, cells were treated with 5 µl MTT reagent/well (5 mg/ml in PBS) and incubated at 37 °C for 1 h. Cells were washed with PBS, and formazan crystals were dissolved in DMSO. Cell survival was measured by reading the plate at 570 nm using a Fluostar Omega plate reader (BMG Lab Tech, Cary, NC). Experiments were repeated three times in triplicates.

### Colony formation assay

HepG2, SK-Hep1, and Hep3B cells (1 × 10^5^) were grown in 6-well plates for 24 h and transfected with EV or TNFAIP8- Myc plasmids for 30 h. Cells were trypsinized, and three thousand cells were re-plated in 6-well plates in triplicate and allowed to grow for 7–10 days. In another experiment, EV transfected or TNFAIP8 stable-expressing HepG2 cells were plated in 6-well plates (3000 cells/well) in triplicate and treated with sorafenib or regorafenib and allowed to grow for 7–10 days. Cells were fixed with cold methanol and stained with 0.5% crystal violet for 30 min. Cells were washed with distilled water and allowed to dry. Blue colonies were counted and plotted. Experiments were repeated at least three times.

### Cyto-ID green fluorescence staining for autophagy determination

To test whether TNFAIP8 induces autophagy in HepG2 and SK-Hep1 cells, cells were grown on coverslips and transfected with EV or TNFAIP8-Myc-tagged plasmid. Another set of cells was pretreated with 3-methyladenine (2 mM) for 18 h and transfected with EV or TNFAIP8-Myc-tagged plasmid for 24 h. Cells were washed with PBS and then stained with Cyto-ID green fluorescence reagents (Enzo Life Sciences, Plymouth Meeting, PA) for 1 h at 37 °C in a cell culture incubator. Cells were washed with PBS, fixed with 4% paraformaldehyde for 15 min, and permeabilized with 0.25% Triton X-100 in blocking buffer (2% BSA (Sigma) in PBS). Permeabilized cells were washed twice with PBS and incubated with 1:500 dilution of anti-Myc rabbit antibody in blocking buffer at 4 °C for 18 h. Cells were then washed twice with PBS and incubated with an Alexa-Fluor-568-conjugated anti-rabbit antibody (Invitrogen) for 1 h at room temperature. Following immunostaining, cells were washed twice with PBS and mounted with Vectashield mounting medium (Vector Lab) containing nuclear DAPI stain. Cells were imaged using an Olympus BX60 fluorescent microscope.

### Oil Red O staining

HCC cells were grown on coverslips and transfected with EV or TNFAIP8 plasmids for 24 h and treated with 100 µM oleic acid (Sigma) for 24 h. Cells were fixed, and Oil Red O (ORO) staining was performed as described previously^18^. After ORO staining, cells were washed with PBS, permeabilized with 0.25% Triton X-100 in blocking buffer (2% BSA in PBS), and incubated with 1:500 dilution of anti-Myc rabbit antibody at 4 °C for 24 h. After a second wash, cells were incubated with an Alexa-Fluor-568-conjugated anti-rabbit antibody (Invitrogen) and mounted with Vectashield mounting medium (Vector Lab) containing nuclear DAPI stain. Cells were imaged using an Olympus BX60 fluorescent microscope attached with the bright field (×40 objective) and photographed.

### Oil Red O staining-based steatosis quantification

HCC cells (1 × 10^4^/well) were grown in 96-well plates in triplicates, transfected with EV or TNFAIP8-Myc plasmids for 24 h, and treated with 100 µM oleic acid for 24 h. After Oil Red O staining, cells were lysed in 100 µl of 1X cell lysis buffer solution (Cell Signaling, Danvers, MA) for 15 min. After gentle shaking, Oil Red O stain released from steatotic cells was transferred to another 96-well plate, and the absorbance at 405 nm was measured using Fluostar Omega plate reader (BMG Lab Tech, Cary, NC) as described previously^19,20^. Experiments were repeated two to three times.

### Molecular modeling and docking of *cis*- and *trans*-oleic acids with mTNFAIP8

The molecular modeling software used for docking and graphic visualization was the Molecular Operating Environment (MOE 2016, Chemical Computing Group, Toronto, Canada). The protein crystal structure of mTNFAIP8 (5jxd.pdb) was evaluated from PDB (https://www.rcsb.org/; accessed date: November 18, 2018) as described previously^16^, and used as the receptor for docking. Default parameters of the MOE docking were employed, and the binding site was defined to be located around the bound ligand in the crystal structure. The binding site surface was generated as a molecular surface based on the pocket atoms.

### ELISA

To test the binding of TNFAIP8 with fatty acids, we developed ELISA. Fatty acids were dissolved in ethanol, and different concentrations of fatty acids such as oleic acid, elaidic acid, palmitic acid, lauric acid, stearic acid, myristic acid, linoleic acid, and cholesterol were coated with hydrophobic lipid-binding ELISA plates (ThermoFisher) and plates were dried by incubation at 37 °C for 2 h. Purified human TNFAIP8-His-tagged protein was obtained from Sino Biological (PA) and anti-TNFAIP8 antibody from Proteintech Group (IL). The purity of TNFAIP8 protein and specificity of TNFAIP8 antibody was examined by Simplyblue staining and immunoblotting of TNFAIP8 protein with TNFAIP8 antibody. Purified equal amounts of TNFAIP8 protein (50 ng) was incubated with different concentrations of fatty acids coated in ELSA plates at 4 °C for 18 h. After three washes, 100 μl of anti-TNFAIP8 antibody (1:500 dilution) was added to each well and further incubated for 18 h at 4 °C. After washing, anti-rabbit-HRP conjugated secondary antibody (1:10,000 dilution) was added to each well for 2 h. After three washes, TMB substrate was added to each well, and the signal was stopped by the addition of stop solution. The solutions were transferred to clear ELISA plates carefully, and the absorbance at 450 nm was measured using Fluostar Omega plate reader (BMG Lab Tech, Cary, NC). Experiments were repeated three times. The signals from vehicle coated wells were subtracted and relative binding affinities of TNFAIP8 toward different fatty acids were determined and plotted.

### Immunoprecipitation

HCC cells (1 × 10^6^/well) were grown in 100 mm dishes and transfected with EV or TNFAIP8-Myc plasmid for 24 h. Cell extracts (1 mg) from EV transfected or TNFAIP8-Myc-transfected were equilibrated in RIPA buffer supplemented with protease inhibitor (Roche Applied Science) and 1 mM PMSF. The lysates were cleared with protein AG plus-agarose beads (Santa Cruz Biotechnology) and incubated with 2 µg of anti-Myc or ATG7 or TNFAIP8 antibody as indicated at 4 °C overnight. Immune complexes were collected by adding 20 µl of protein AG-agarose beads and after washing (three times) with RIPA buffer, and proteins were resolved on a 4–12% Bis-Tris SDS-PAGE and immunoblotted with indicated antibodies.

### Identification of TNFAIP8-ATG7 interaction by mass spectrometry

PC3 cell extracts (2 mg) were IPed with control rabbit IgG or anti-TNFAIP8 antibody at 4 °C overnight. Immune complexes were collected by adding 20 µl of protein AG-agarose beads and after washing (three times) with RIPA buffer, and proteins were resolved on a 4–12% Bis-Tris SDS-PAGE and gel was stained with Simplyblue. The gel was excised into six sections, digested with trypsin, and the proteins were identified by comparing the mass spectra (*m*/*z*) from the human sequence databases using MS Proteomics core facility at Yale University (https://medicine.yale.edu/keck/proteomics/).

### Animal care and treatment

All animal procedures were carried out in accordance with the NIH Guidelines for the Care and Use of Laboratory Animals and approved by the NCCU Institutional Animal Care and Use Committee (NCCU-IACUC Protocol No. is MG-02-26-2010). All the techniques proposed for use on animals are included in an IACUC-approved protocol. Every effort was made to assure the comfort and safety of the animals. Liquid diets, purchased from DYETS Inc (Bethlehem, PA), were based upon the Lieber-DeCarli EtOH formulation and provided 1 kcal/mL. Ten-week-old male C57BL/6J mice were ear tagged and randomly assigned to one of two groups and either pair-fed a control diet (*n* = 6) or a standard Lieber-Decarli liquid diet, containing 5% EtOH (*n* = 6) (representing 27.5% of the total caloric intake), for 8 weeks as previously described^21^. In another experiment, mice were fed either a chow diet (*n* = 4) (control diet, 12% calories as fat) or a high-fat diet (*n* = 4) (HFD, 45% calories as fat) for 16 weeks. Our pre-established inclusion/exclusion criteria were that animals will be excluded from the analysis, if they were too sick or died before the end of the study. After 8 weeks of EtOH or 16 weeks high-fat diet feeding, mice were anesthetized with isoflurane and sacrificed, and liver sections were rapidly dissected, weighed, snap-frozen in liquid nitrogen and kept at −80 °C. Liver slices were also fixed in 10% formalin/phosphate-buffered saline, and liver sections were prepared. The sections were stained with H&E for histological examination. Liver tissue extracts were homogenized and prepared as described previously^21^. The rationale for the sample size was based on our published reports^21,22^ indicating that 4–6 mice are needed to detect a significant difference in triglyceride accumulation between the experimental groups and their littermate control, with alpha error of 0.05 and power of test of 0.8. Therefore, a sample size of 4–6 is often sufficient to detect changes in lipid metabolism, and steatosis in both chronic EtOH and the HFD models we used in this report. All data set exhibited normal distribution.

### Statistical analysis

Results are from independent duplicate or triplicate experiments and presented as means ± SEM. Differences between groups were analyzed using either two-tailed Student’s *t*-test or one-way ANOVA followed by Tukey HSD post hoc test. A *P* value of <0.05 was considered statistically significant. Statistical analyses were performed using the IBM SPSS Statistics 25 software (Armonk, NY).

## Results

### Higher TNFAIP8 expression associated with liver cancer in human patients

**Fig. 1:**
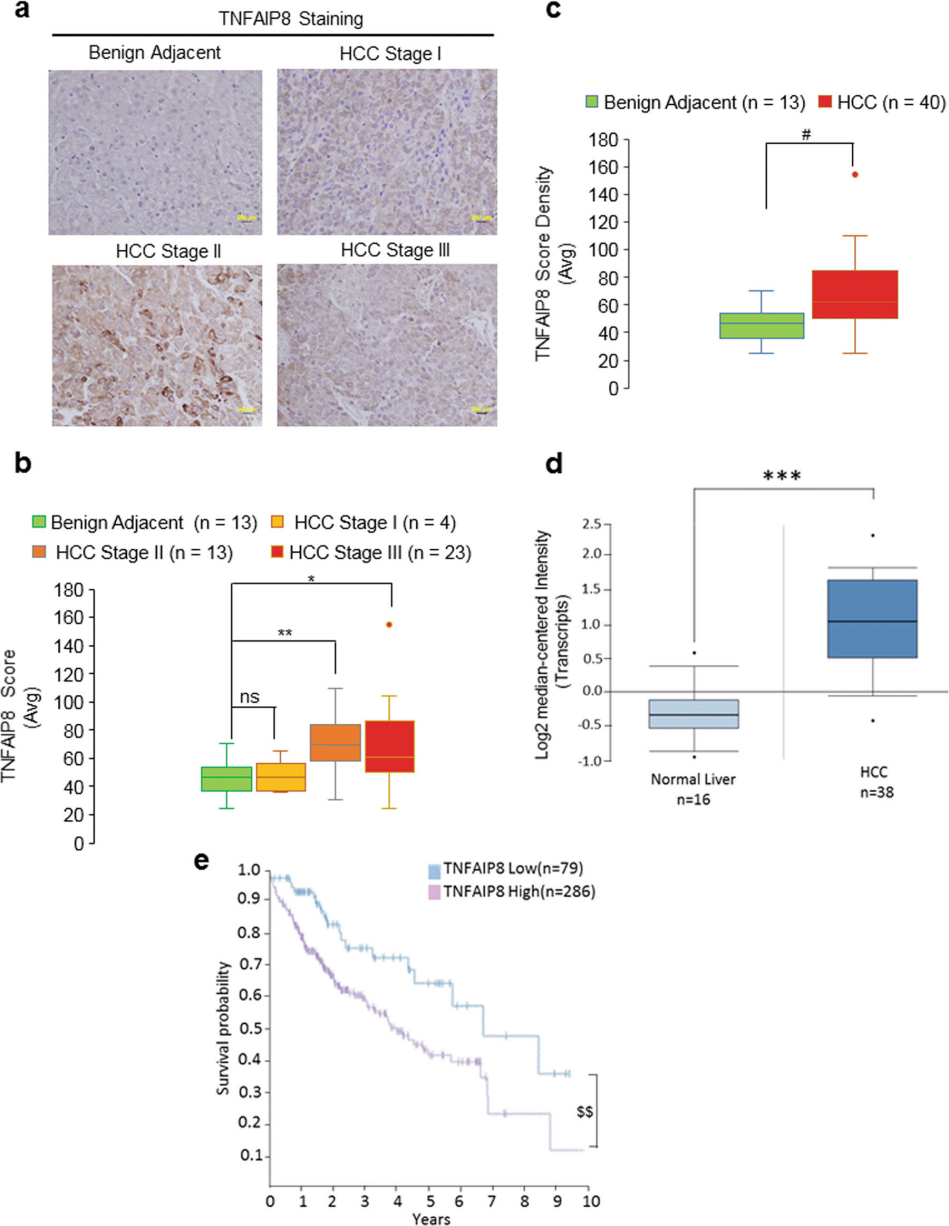
Higher TNFAIP8 expression is associated with liver cancer progression. **a** Representative images of tumor microarray (TMA) of TNFAIP8 expression in different stages of liver cancer. **b** Expression levels of TNFAIP8 protein in normal livers, stage I, stage II, and stage III HCC tumors were quantified from TMA. Boxplots show median and interquartile ranges with whiskers indicating the total range of TNFAIP8 expression. The data represent mean from 4 to 23 HCC tissues. *P < 0.05, **P < 0.01, ns—not significant relative to benign adjacent tissues. **c** The overall quantifi**c**ation of TNFAIP8 expression in benign adjacent liver tissues and HCC tumors with different stages was quantified from TMA. Boxplots show median and interquartile range with whiskers indicating the total range of TNFAIP8 expression. The data represent mean from 13 to 40 HCC tissues. ^#^*P* < 0.05 statistical significance relative to benign adjacent tissues. **d** Expression of TNFAIP8 transcripts between normal liver and HCC tumors were presented from Oncomine data set. (https://www.oncomine.org/resource/login.html; accessed October 7, 2019). ***P < 0.001 compared with normal liver. **e** Effect of low and high TNFAIP8 expression and related liver cancer patient survival probabilities reported in ‘The Human Protein Atlas’ was presented (https://www.proteinatlas.org/search/TNFAIP8; accessed November 12, 2018). ^$$^*P* < 0.01 statistical significance relative to low TNFAIP8 expression.

We performed immunohistochemical staining to assess TNFAIP8 protein expression levels in different stages of liver cancer (Fig. 1a–c). Tissue microarray (TMA) data revealed that TNFAIP8 expression was significantly higher in stage II and stage III liver tumor tissues (Fig. 1a, b) and the overall TNFAIP8 expression was significantly higher in liver tumors (*n* = 40) compared with benign adjacent liver tissues (*n* = 13) (Fig. 1c). Our data are further supported by Oncomine data which reveals that expression of TNFAIP8 transcripts is 2.418-fold higher in liver tumors compared with normal liver tissues (Fig. 1d) (https://www.oncomine.org/resource/login.html accessed October 7, 2019). In addition, the data from Human Protein Atlas demonstrated that a higher expression of TNFAIP8 in liver tumors significantly reduced (21%) the 5-year survival rate of liver cancer patients compared with TNFAIP8-lower-expressing liver tumors suggesting that high expression of TNFAIP8 in liver tumors is associated with poor prognosis in liver cancer patients (Fig. 1e) (https://www.proteinatlas.org/search/TNFAIP8; accessed November 12, 2018).

To further investigate the role of TNFAIP8 in liver cancer, we analyzed the expression of TNFAIP8 mRNA and protein levels in four HCC cell lines (Supplementary Fig. 1a, b). Higher expression of TNFAIP8 mRNA and protein levels were observed in Hep3B and HepG2 cells compared with SK-Hep1 and PLC/PRF/5 cells (Supplementary Fig. 1a, b). *TNFAIP8* gene is expressed in several isoforms/variants in cancer^12^. By using isoform-specific primers, we demonstrated that HepG2, SK-Hep1, and Hep3B cells expressed predominantly the TNFAIP8 isoform 2 (variant 2) (Supplementary Fig. 1c, d). Involvement of TNFAIP8 variant 2 in lung cancer development and progression has been reported earlier^23^. The expression of TNFAIP8 variant 1 was detected in HepG2 and Hep3B cells but not in SK-Hep1 cells, and isoforms three, four, and five were not detected in any of the cell lines (Supplementary Fig. 1d). Thus, variants 1 and/or 2 appear to account for the majority of the effects we observe in these cell lines. Any differences in the functional roles between isoforms have not yet been delineated.

### TNFAIP8 induces cell survival/drug resistance in HCC cells by inhibiting apoptosis

**Fig. 2:**
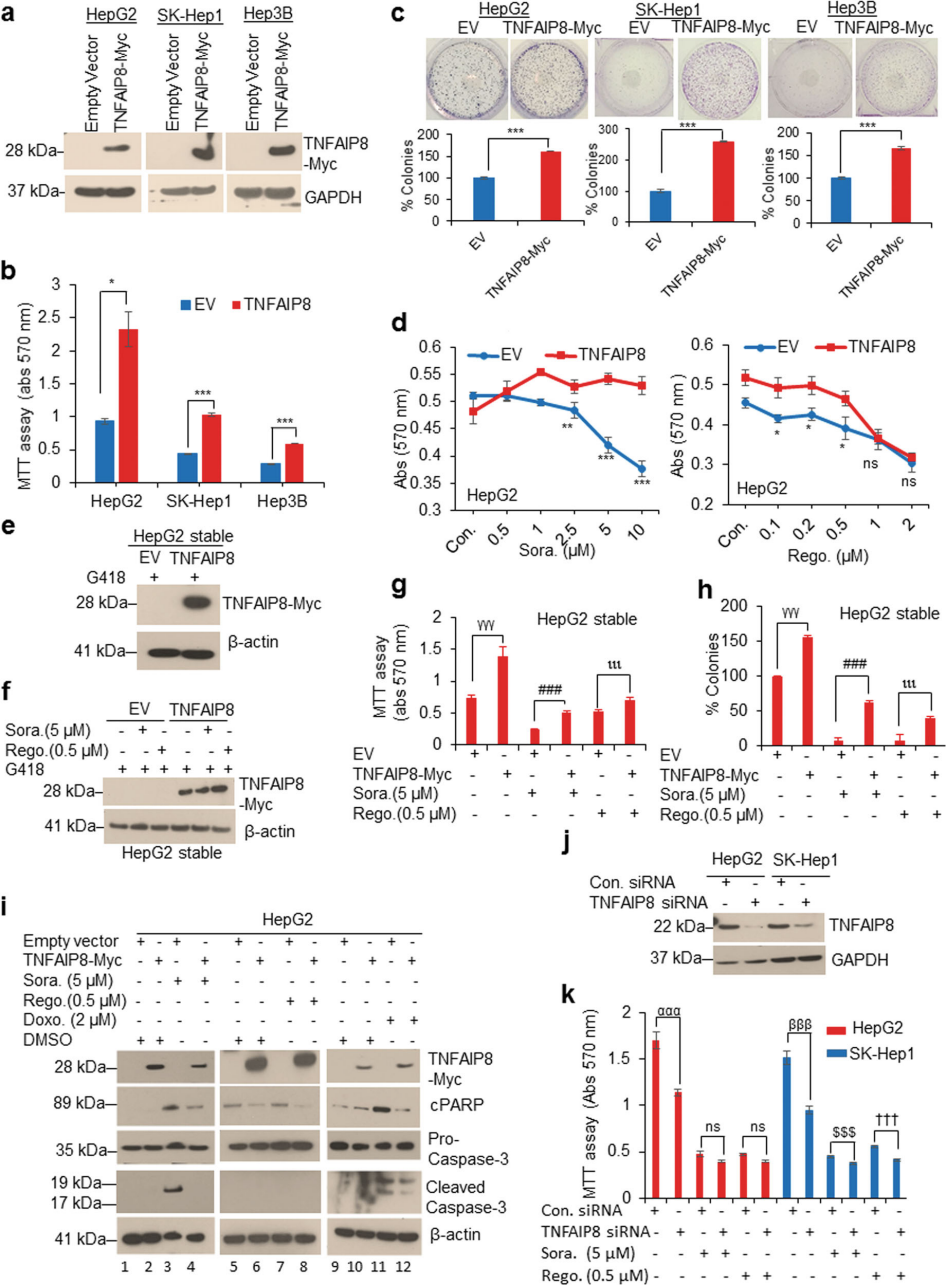
TNFAIP8 induces liver cancer cell survival and drug resistance by reducing apoptosis. **a** WB analysis of TNFAIP8-Myc tagged protein expression in HCC cells. **b** Effect of overexpression of TNFAIP8 on HCC cell survival was measured by MTT assay. **c** Effect of overexpression of TNFAIP8 on liver cancer cell colony formation. **d** Effects of sorafenib or regorafenib on HepG2 cell survival transfected with EV or TNFAIP8-Myc-tagged plasmids were measured by MTT assay. **e** Stable expression of TNFAIP8 in HepG2 was analyzed by WB. **f** EV and TNFAIP8-Myc-stable-expressing cells were treated with sorafenib (5 µM) or regorafenib (0.5 µM) for 48 h, and expression of TNFAIP8-Myc tagged protein was analyzed by WB. **g**, **h** Effects of sorafenib (5 µM) or regorafenib (0.5 µM) on EV and TNFAIP8-Myc-stable-expressing HepG2 cells on survival and cell colony formation were measured. **i** WB analysis of the effect of sorafenib, regorafenib, and doxorubicin on cell apoptosis markers expression in HepG2 cells transfected with EV or TNFAIP8-Myc-tagged plasmids. **j** HepG2 and SK-Hep1 cells were transfected with control siRNA or TNFAIP8 siRNA for 30 h, and cell lysates were WB with anti-TNFAIP8 and anti-GAPDH antibodies. **k** Control siRNA and TNFAIP8-siRNA transfected cells (10,000) were re-plated in 96-well plates and treated with sorafenib (5 µM) or regorafenib (0.5 µM) for 48 h, and cell survival was measured by MTT assay. Data represent mean ± SEM from two to three independent experiments. *P < 0.05, **P < 0.01, ***P < 0.001 relative to EV transfected, and Sora or Rego-treated. ^γγγ^*P* < 0.001 compared with EV stably transfected. ^###^*P* < 0.001 relative to EV stably transfected and Sora treated. ^ιιι^*P* < 0.001 compared with EV transfected and Rego-treated. ^ααα^*P* < 0.001, ^βββ^*P* < 0.001 compared with control siRNA transfected. ^$$$^*P* < 0.001 compared with control siRNA transfected and Sora treated. ^†††^*P* < 0.001 relative to control siRNA transfected and Rego-treated. EV: empty vector, Sora: sorafenib, Rego: regorafenib, NS: not significant, WB: western blotting.

The effect of TNFAIP8 on HCC cell survival, drug resistance, and apoptosis was determined in HCC cells. Overexpression of TNFAIP8-Myc tagged protein increased cell survival and cell colony formation (Fig. 2a–c). To examine the effect of TNFAIP8 on drug resistance, TNFAIP8-transfected cells were treated with increasing concentrations of two anti-liver cancer drugs, sorafenib, and regorafenib (Fig. 2d). Dose-dependent treatment with sorafenib (0.5–10 µM) decreased cell survival in empty-vector-transfected cells, whereas overexpression of TNFAIP8 resulted in significant resistance (Fig. 2d, left panel). Overexpression of TNFAIP8 also caused significant resistance in cells treated with a low concentrations regorafenib (0.1–0.5 µM) but was unable to cause significant resistance in cells treated with high concentrations of regorafenib (1–2 µM) (Fig. 2d, right panel). Similarly, stable expression of TNFAIP8 in HepG2 cells significantly attenuated the effects of sorafenib (5 µM) and regorafenib (0.5 µM) on cell survival and cell colony formation (Fig. 2e–h). We also examined the role of TNFAIP8 in drug-mediated apoptosis (Fig. 2i). Treatment with sorafenib, regorafenib, and doxorubicin induced cleaved PARP (cPARP) expression in EV transfected HepG2 cells (Fig. 2i, lanes 3, 7 & 11), but was significantly reduced when cells were transfected with TNFAIP8 (Fig. 2i, lanes 4, 8 & 12). Cleaved caspase-3 was also increased in sorafenib and doxorubicin treated EV transfected cells but decreased in TNFAIP8 and drug-treated cells (Fig. 2i, lanes 3, 4 & 11, 12). No cleaved caspase-3 expression was detected in regorafenib treated cells, but increased expression of pro-caspase-3 was observed in TNFAIP8-transfected cells treated with regorafenib (Fig. 2i, lane 8). Treatment with sorafenib or regorafenib also significantly decreased endogenous TNFAIP8 protein levels in HepG2 and SK-Hep-1 cells and induced cPARP expression in HepG2 cells compared with vehicle-treated cells (Supplementary Fig. 2a). No significant change in TNFAIP8 mRNA levels was observed in HepG2, and SK-Hep1 cells treated with regorafenib, whereas downregulation of the expression of TNFAIP8 mRNA was observed in sorafenib-treated (5 µM) SK-Hep1 cells (Supplementary Fig. 2b). In addition, TNFAIP8 knockdown by siRNA decreased cell survival by 30–40% in both cell lines (Fig. 2j, k). Combination of TNFAIP8 knockdown and sorafenib (5 µM) or regorafenib (0.5 µM) treatment reduced cell survival by 18–24%, compared with drug-treated SK-Hep1 cells and transfected with control siRNA. The data also indicated that TNFAIP8 knockdown in HCC cells increased sensitivity (~10-fold) to regorafenib-induced cell death compared with sorafenib (Fig. 2k). Collectively, these data suggest that TNFAIP8 increases cell survival and drug resistance and decreases apoptosis in HCC cells.

### TNFAIP8 promotes autophagy in HCC cells

**Fig. 3:**
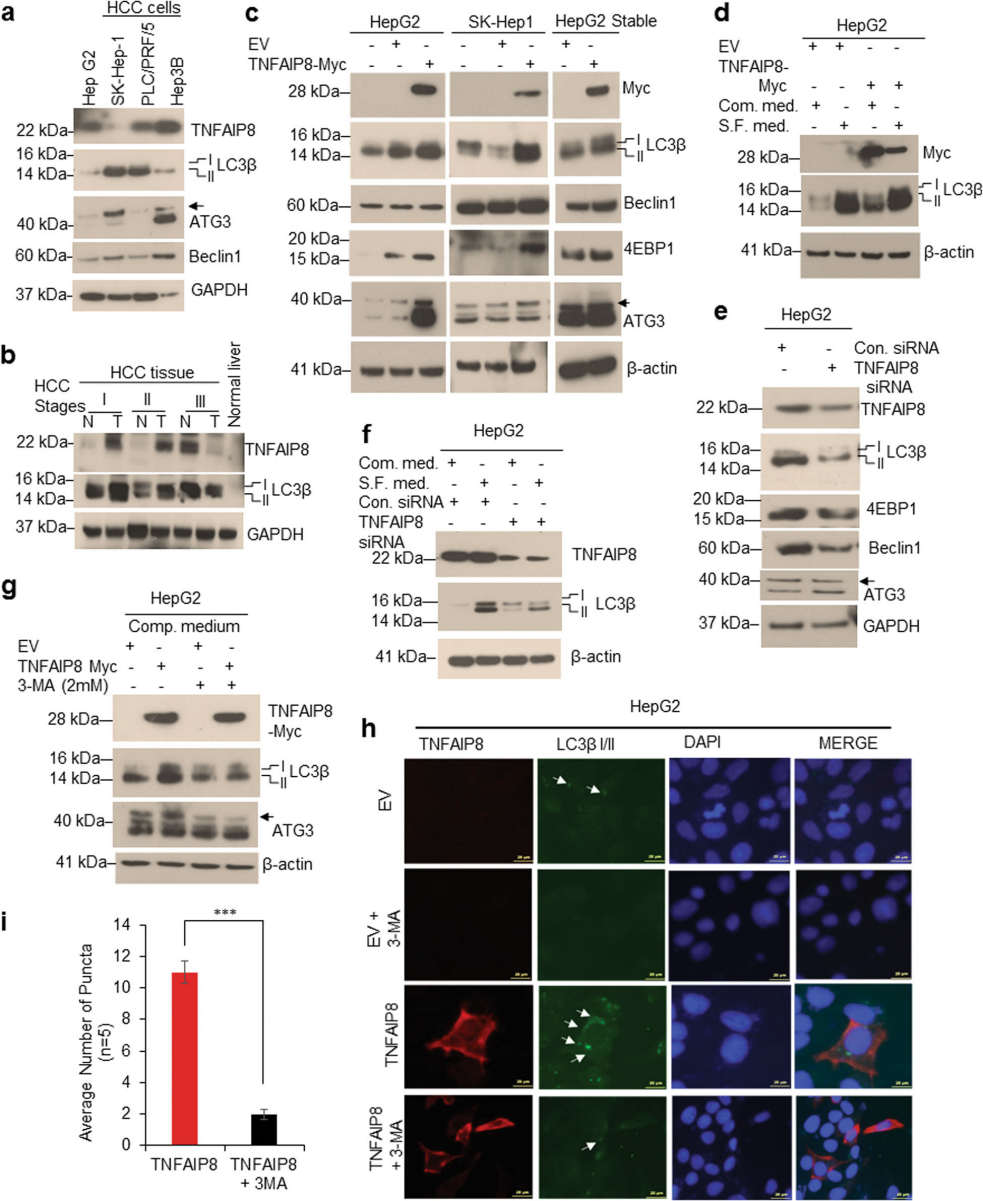
TNFAIP8 induces autophagy in liver cancer cells. **a** Fifty micrograms of lysates from HCC cells immunoblotted with indicated antibodies. **b** Fifty micrograms liver cancer tissue lysates with different stages of liver cancer were immunoblotted with indicated antibodies. **c** HepG2, SK-Hep1 cells were transfected with EV or TNFAIP8-Myc plasmid for 30 h, and cell lysates from transient and stable HepG2-TNFAIP8 expressing cells were immunoblotted with indicated antibodies. **d** HepG2 cells were grown in complete medium or serum-free (S.F.) medium for 24 h and then transfected with EV or TNFAIP8-Myc plasmid for 30 h. Fifty micrograms of proteins were immunoblotted. **e** HepG2 cells were transfected with control siRNA, or TNFAIP8 siRNA (100 nM) for 30 h and lysates were immunoblotted with autophagy related bio-markers. **f** HepG2 cells were grown in complete medium or serum-free (S.F.) medium for 24 h and then transfected with control siRNA or TNFAIP8 siRNA (100 nM) for 30 h as indicated, and lysates were immunoblotted with indicated antibodies. **g** HepG2 cells were pretreated with the autophagy inhibitor, 3-MA for 18 h and transfected with EV and TNFAIP8-Myc plasmid for 24 h in the presence of 3-MA and cell lysates were immunoblotted with indicated antibodies. **h**, **i** Effect of TNFAIP8 overexpression and 3-MA on LC3β related puncta formation in HepG2 cells were analyzed by Cyto-ID green fluorescence staining (**h**), and number of puncta were measured from TNFAIP8-transfected or 3-MA pretreated and TNFAIP8-transfected cells and plotted (**i**). ***P < 0.001 relative to TNFAIP8 transfected. EV: empty vector, Comp. medium: complete medium, S.F. medium: serum-free medium, N: normal, T: tumor. Arrow indicates ATG3 specific band.

In an earlier study, we demonstrated that TNFAIP8 induces autophagy in prostate cancer cells^12^. Here we investigated whether TNFAIP8 modulates autophagy in liver cancer cells. First, we analyzed the endogenous levels of TNFAIP8 and autophagy markers expression in four HCC cell lines. Under basal conditions, there was no correlation found between endogenous TNFAIP8 expression and autophagy bio-markers LC3β I/II, ATG3, and Beclin-1 expression (Fig. 3a). We also analyzed the expression of TNFAIP8 and LC3β I/II in different stages of liver cancer tumors. Interestingly, higher expression of TNFAIP8 and LC3β I/II was found in stage I and stage II liver cancer tumors compared with adjacent liver tissues or normal liver tissues (Fig. 3b). To clarify whether TNFAIP8 expression in HCC cells associated with autophagy induction, we overexpressed the TNFAIP8-Myc-tagged protein transiently or stably in HCC cells (Fig. 3c). Ectopic expression of TNFAIP8 induced expression of LC3β I/II, ATG3, 4EBP1, and Beclin-1 (Fig. 3c). Serum starvation is known to induce autophagy in many cancer cells, including HCC cells^24^. Serum-starved HepG2 cells showed increased expression of LC3β I/II, compared with cells grown in complete medium. Likewise, both ectopic expression of TNFAIP8 alone and a combination of serum-starvation and TNFAIP8 expression induced LC3β I/II expression (Fig. 3d). In contrast, TNFAIP8 knockdown reduced the expression of LC3β I/II, 4EBP1, Beclin-1, and ATG3 autophagy bio-markers (Fig. 3e) and serum-starvation-induced expression of LC3β I/II was significantly reduced in TNFAIP8-knockdown HepG2 cells (Fig. 3f). Pre-treatment with autophagy inhibitor, 3-methyladenine (3-MA) reduced the TNFAIP8-mediated expression of LC3β I/II and ATG3 proteins (Fig. 3g), LC3β I/II-related puncta formation (arrows) in HepG2 (Fig. 3h, i) or in SK-Hep1 (Supplementary Fig. 2c) or HepG2-TNFAIP8-stable expression cells (Supplementary Fig. 2d), suggesting that TNFAIP8 induces autophagy in HCC cells.

### TNFAIP8 interacts with ATG7-ATG3 complex

**Fig. 4:**
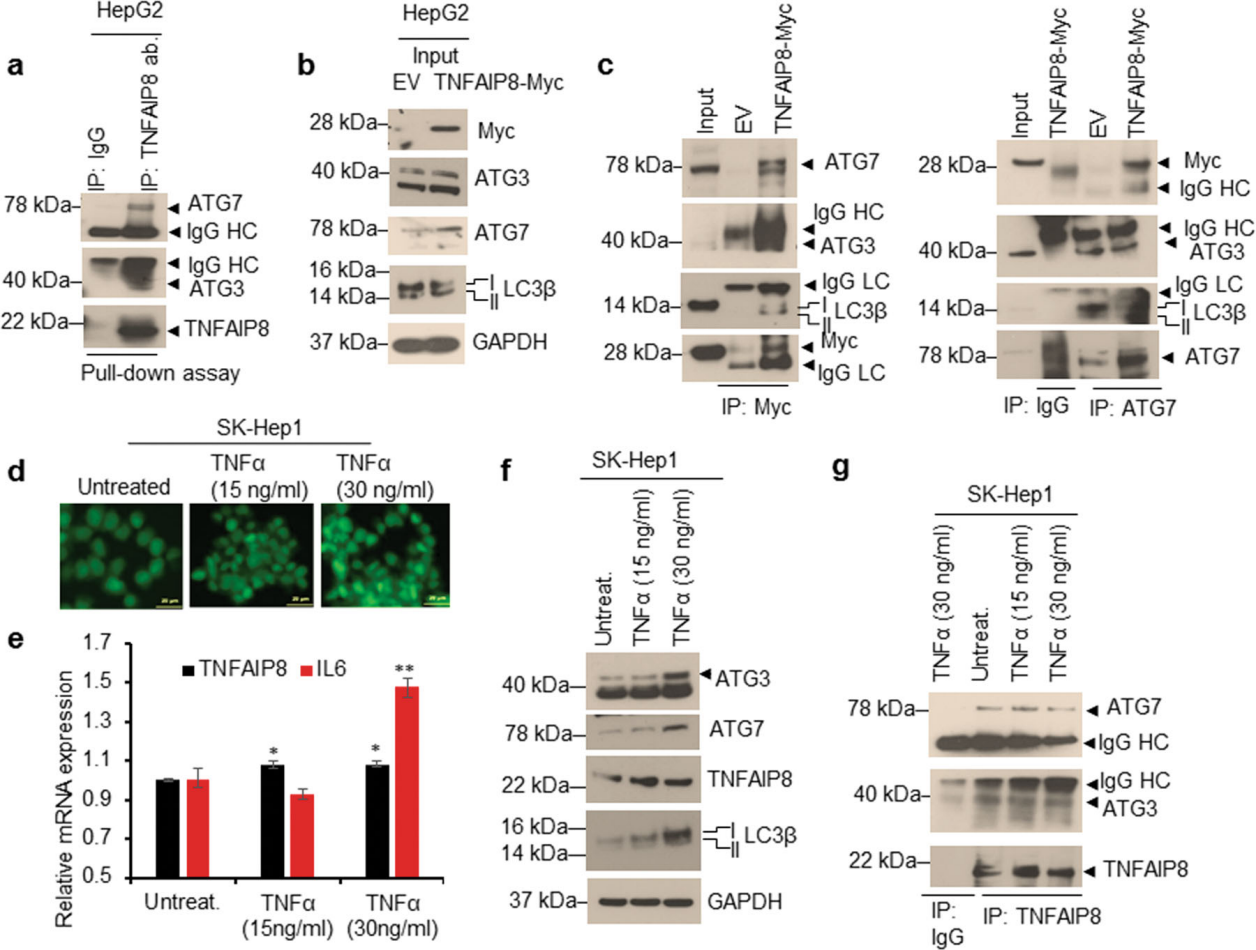
TNFAIP8 interacts with ATG7-ATG3 complex. **a** HepG2 cell lysates (1 mg) were incubated with pure TNFAIP8 protein (2 µg) for 3 h. TNFAIP8 was pulled-down with TNFAIP8 antibody or control IgG. Immunocomplexes were resolved by SDS-PAGE and WB with indicated antibodies. **b**, **c** Interaction of TNFAIP8-Myc protein with ATG7-ATG3-LC3B complex was analyzed by forward and reverse IP as indicated. **d** Effect of TNFα on cellular ROS production was visualized by staining the cells with CellROX Green reagent (Invitrogen). **e** Effect of TNFα on TNFAIP8 and IL-6 mRNA expression was analyzed by RT/qPCR (*n* = 3). **f**, **g** Effect of TNFα on ATG3, ATG7. TNFAIP8 and LC3B protein expression were analyzed by WB and interaction of TNFAIP8 with ATG3-ATG7 was analyzed by TNFAIP8 IP followed by WB. *P < 0.05, **P < 0.01 relative to untreated SK-Hep1 cells. IP: immunoprecipitation, WB: western blotting.

Since TNFAIP8 induced autophagy, we asked whether TNFAIP8 interacts with autophagosome components. We immunoprecipitated (IPed) endogenous TNFAIP8 protein from PC3 cell lysates and TNFAIP8 interacting proteins were identified by Mass Spectrometry (Supplementary Fig. 3a, b). LC-MS analysis suggests that TNFAIP8 specifically interacts with autophagy-related protein 7 (ATG7). Previously we and others demonstrated that TNFAIP8 interacts with autophagy-related protein 3 (ATG3)^12,25^. To validate these interactions, we incubated purified TNFAIP8 protein with HepG2 cell lysates, and TNFAIP8 protein was immunoprecipitated with TNFAIP8 antibodies. The pull-down assay demonstrated that TNFAIP8 interacts with both ATG7 and ATG3 (Fig. 4a). Also, using forward and reverse IP followed by WB, we confirmed that TNFAIP8-Myc tagged protein expressed in HepG2 cells specifically interacts with ATG7-ATG3 and LC3 (Fig. 4b, c). Since cytokine TNFα upregulates cellular inflammation and TNFAIP8 expression^4^, and a recent report suggested that oxidation of critical thiols of ATG7 and ATG3 inhibits autophagy^26^, we further analyzed the effect of cellular inflammation induced by TNFα on TNFAIP8 protein expression and TNFAIP8 interaction with ATG7 and ATG3 proteins (Fig. 4d–g). Exposure of TNFα (30 ng/ml) significantly increased ROS production (Fig. 4d) and TNFAIP8 and inflammatory marker Interleukin-6 (IL-6) mRNA expression in SK-Hep-1 cells (Fig. 4e). Treatment of TNFα (30 ng/ml) also increased TNFAIP8, ATG3, ATG7, and LC3β II protein expression (Fig. 4f). Induction did not alter ATG7-ATG3 interaction with TNFAIP8 compared with untreated or 15 ng/ml TNFα treated cells (Fig. 4g).

### TNFAIP8 inhibits AKT/mTOR signaling and promotes autophagy-mediated cell survival

**Fig. 5:**
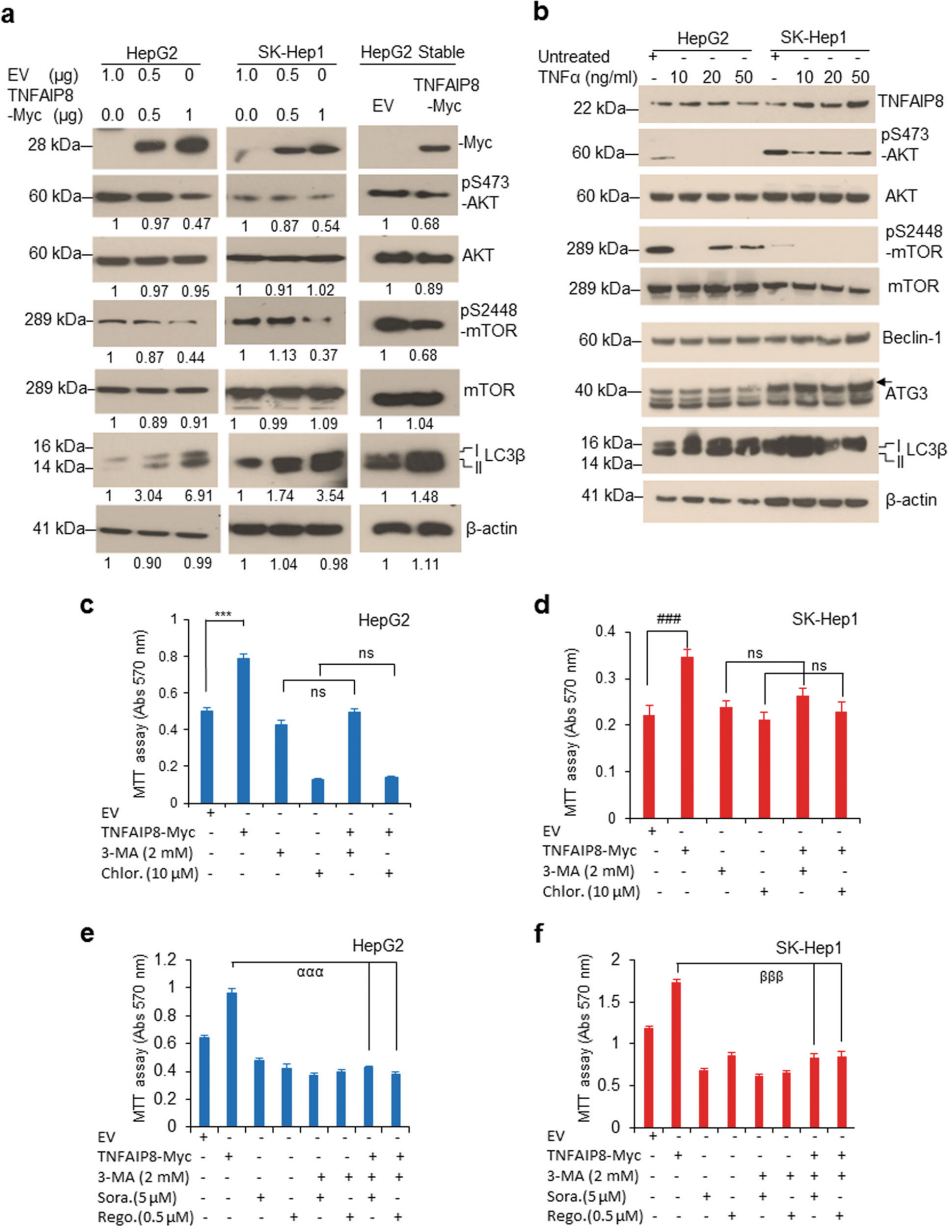
TNFAIP8 inactivates AKT/mTOR and induces autophagy. **a** Cell lysates from HepG2 and SK-Hep1 cells transfected with EV and TNFAIP8-Myc plasmids (left and middle panels) and EV or TNFAIP8-Myc-stable-expressing HepG2 cells (right panel) were WB with indicated antibodies. Indicated protein levels were quantified using ImageJ software (https://imagej.nih.gov/ij/). **b** HCC cells were treated with TNFα (10 to 50 ng/ml) for 30 h and lysates were WB with indicated antibodies. **c**, **d** HepG2 and SK-Hep1 cells were pretreated with 3-MA (2 mM) or chloroquine (10 µM) for 8 h and cells were transfected with EV or TNFAIP8-Myc plasmid in the presence of the indicated autophagy inhibitor for 40 h, and cell survival was measured by MTT assay. **e**, **f** HepG2 and SK-Hep1 cells were pretreated with 3-MA (2 mM) for 8 h, the cells were transfected with EV or TNFAIP8-Myc plasmid in the presence of autophagy inhibitor for 24 h and then treated with sorafenib (5 µM) or regorafenib (0.5 µM) for additional 40 h, and cell survival was measured by MTT assay. Data represent mean ± SEM  from four (**d**–**f**) independent experiments. ***P < 0.001, ^###^*P* < 0.001 compared with EV transfected. ^ααα^*P* < 0.001, ^βββ^*P* < 0.001 compared with TNFAIP8 transfected. EV: empty vector, NS: not significant, 3-MA: 3-methyladenine, Chlor: chloroquine, Sora: sorafenib, Rego: regorafenib.

The AKT/mTOR pathway is known to control cellular autophagy^27,28^. Therefore, we examined whether TNFAIP8 regulates AKT/mTOR signaling and autophagy modulation in HCC cells. Transient or stable expression of TNFAIP8 in HCC cells significantly reduced the phosphorylation of Serine-473 of AKT (pS473-AKT) and Serine-2448 phosphorylation of mTOR (pS2448-mTOR) (Fig. 5a). On the other hand, TNFAIP8 induced expression of LC3β I/II in all three HCC cell lines (Fig. 5a). To further support this, we induced endogenous TNFAIP8 expression by exposing the HCC cells to cytokine TNFα. Dose-dependent treatment of TNFα (10–50 ng/ml) increased expression of endogenous TNFAIP8, whereas, the expression of autophagy biomarker LC3β I/II was increased at low concentration of TNFα (10–20 ng/ml) and decreased at higher concentrations (50 ng/ml). Interestingly, TNFα treatments (10–20 ng/ml) significantly decreased the expression of pS473-AKT and pS2448-mTOR in both cell lines (Fig. 5b), suggesting that TNFα mediated induction of TNFAIP8 inhibits AKT/mTOR. The observed effects on the AKT/mTOR pathway are consistent with TNFAIP8-induced autophagy.

To test whether TNFAIP8 induces cell survival/drug resistance by the induction of autophagy, HCC cell lines were pretreated with the autophagy inhibitors, 3-MA or chloroquine, and transfected with TNFAIP8 (Fig. 5c, d). Expression of TNFAIP8 significantly increased cell survival in both cell lines. However, when cells were pretreated with the autophagy inhibitors, 3-MA, and chloroquine, TNFAIP8 was unable to promote cell survival (Fig. 5c, d). Moreover, inactivation of autophagy by 3-MA and treatment with anti-liver cancer drugs sorafenib or regorafenib in HCC cells significantly prevented TNFAIP8-mediated cell survival (Fig. 5e, f), suggesting that TNFAIP8-mediated induction of autophagy is required for cell survival and drug resistance in HCC cells.

### TNFAIP8 binds oleic acid and increases cell steatosis

**Fig. 6:**
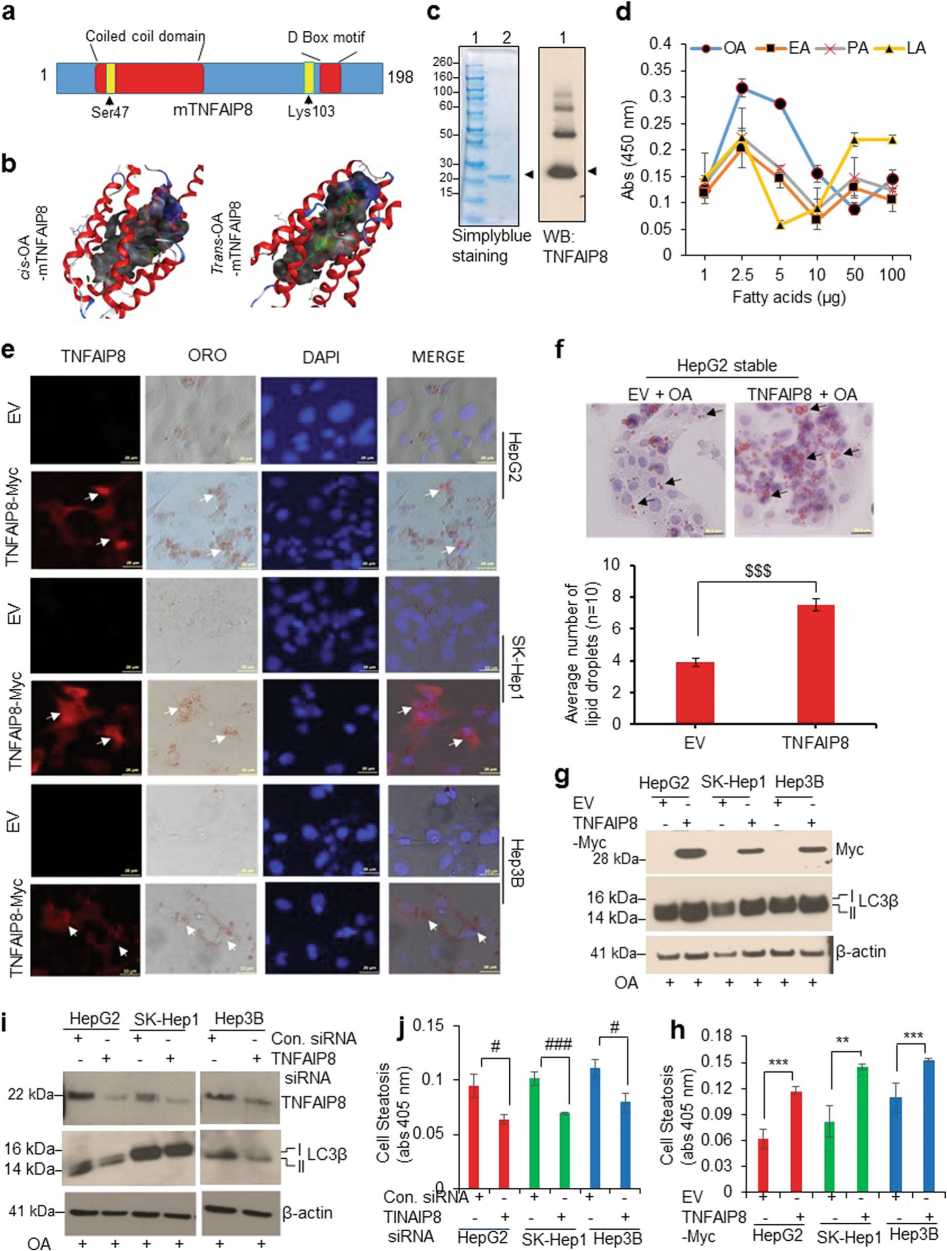
TNFAIP8 binds with oleic acid and enhances HCC cell steatosis. **a** The location of the coiled-coil domain, D-Box consensus, and the positions of *cis*-/*trans*-OA binding key amino acids of TNFAIP8 are shown. **b** Molecular modeling images of *cis*- and *trans*-oleic acid binding with mouse TNFAIP8 are shown (left and right panels). **c** The purity of TNFAIP8-His-tagged protein and specificity of TNFAIP8 antibody was examined by simplyblue staining and immunoblotting. **d** Binding of TNFAIP8 with fatty acids was determined by ELISA as described in materials and methods section. OA: oleic acid, EA: elaidic acid, PA: palmitic acid, LA: lauric acid. **e** HCC cells were grown on coverslips and transfected with EV or TNFAIP8-Myc plasmid and treated with 100 µM oleic acid for 24 h. Cells were stained with Oil Red O (ORO) and anti-Myc rabbit antibody overnight, followed by Alexa-Fluor-568-conjugated anti-rabbit secondary antibody. After washing, cells were stained with DAPI, mounted, and photographed (arrow indicate lipid droplets in TNFAIP8 overexpressing cells). **f** EV and TNFAIP8-Myc-stable-expressing HepG2 cells were grown on coverslips and treated with 100 µM oleic acid for 24 h and stained with ORO (upper panel). The number of ORO-stained lipid droplets present in the EV and TNFAIP8-stable-expressing cells was measured and plotted (lower panel). **g** HCC cells were grown in 6-well plates and transfected with EV or TNFAIP8-Myc plasmids for 30 h and treated with 100 µM oleic acid for 24 h and lysates were WB with indicated antibodies. **h** Similarly, transfected and treated cells were stained with ORO, cells were lysed, and ORO stain released from steatotic cells was measured and plotted. **i** HCC cells were transfected with control siRNA or TNFAIP8 siRNA for 30 h and lysates were WB with indicated antibodies. **j** Control siRNA or TNFAIP8-siRNA transfected cells were treated with 100 µM oleic acid for 24 h, and after Oil Red O staining, cells were lysed, and ORO stain released from steatotic cells was measured and plotted. All data represent mean ± SEM from two (**d**, **j**, **h**) independent experiments in triplicate. ^$$$^*P* < 0.001 relative to EV transfected. **P < 0.01, ***P < 0.001 relative to EV transfected and OA treated cells. ^#^*P* < 0.05, ^###^*P* < 0.001 relative to control siRNA transfected cells. EV: empty vector, OA: oleic acid.

We recently analyzed the molecular structure of human TNFAIP8 protein using a molecular modeling approach^4^. Similar to the mTNFAIP8 crystal structure^16^, the predicted structure of hTNFAIP8 exhibited a large cylindrical central hydrophobic deep cavity surrounded by seven cylindrical helices. The coiled-coil structural motif was localized to α helices 2 and 3, and the D-Box motif was present in α helix 5 (Fig. 6a, b). Earlier, Kim et al.^16^ suggested that phospholipids and fatty acids, particularly oleic acid (OA)-like substrates, can bind with TNFAIP8 proteins. Since hTNFAIP8 is structurally similar to mTNFAIP8 (94.4% amino acid similarity), we used a molecular modeling approach to better understand the binding of *cis*- and *trans*-OA with mTNFAIP8. Molecular modeling data indicated that both *cis* and *trans* forms of OA bind with mTNFAIP8 in the deep hydrophobic cavity (Fig. 6b). Docking of the *cis*-isomer (i.e., oleic acid) suggests that the oxygen atoms of the carboxyl group form hydrogen bonds with the hydroxy group (–OH) of N-terminal Ser^47^-mTNFAIP8, which was localized to helix α2 as part of the coiled-coil (CC) domain. Hydrogen bond distances are 3.22 and 3.83 Å, respectively (Fig. 6b, left panel). On the other hand, docking of the *trans*-isomer (i.e., elaidic acid) indicates that the oxygen atoms of the carboxyl group form hydrogen bonds with the amino group (–NH_2_) of Lys^103^-mTNFAIP8, which is localized in helix α4 packing against helix α5 near to D-Box motif (Fig. 6b, right panel), suggesting that both *cis*- and *trans*-OA bind with mTNFAIP8. Also, we validated the binding profile of several fatty acids using ELISA (Fig. 6c, d). ELISA demonstrated that TNFAIP8 specifically binds to oleic acid, elaidic acid, palmitic acid, and lauric acid (Fig. 6d) and myristic acid to a lesser extent. However, no binding of TNFAIP8 with stearic acid, linoleic acid, or cholesterol was observed (Supplementary Fig. 4a). Further, when we exposed HCC cells to *cis-* and *trans-*OA, intracellular lipid droplet formation (steatosis) was observed, indicating that both *cis-* and *trans-*OA induce cell steatosis to approximately the same extent in HCC cells (Supplementary Fig. 4b) and exposure of *cis-* and *trans-*OA stabilized endogenous TNFAIP8 and increased expression of autophagy biomarker LC3β I/II (Supplementary Fig. 4c). To test whether TNFAIP8 induces OA-mediated cell steatosis, we overexpressed TNFAIP8-Myc tagged in HCC cells and treated with OA. As shown in Fig. 6e, TNFAIP8-Myc tagged overexpressing HCC cells showed a specific increase in oleic-acid-mediated cell steatosis (lipid droplet formation) (Fig. 6e). Increased numbers (~2-fold) of oleic-acid-mediated intracellular lipid droplets were also observed in TNFAIP8 stable expressing HepG2 cells (Fig. 6f, upper and lower panels). Overexpression of TNFAIP8 combined with OA treatment also induced the expression of LC3β I/II in all three indicated HCC cell lines and quantitation of ORO-based steatosis (lipid content) suggests that TNFAIP8 expression in HCC cells increased oleic acid uptake/accumulation by ~1.5–2.1-fold in HCC cells (Fig. 6g, h). On the other hand, TNFAIP8 knockdown by siRNA and treatment with OA downregulated the expression of the autophagy marker, LC3β I/II (Fig. 6i) and significantly reduced intracellular cell steatosis (lipid content) (Fig. 6j) and oleic acid-mediated lipid droplet formation (Supplementary Fig. 5a, b).

Since numerous lipid and fatty-acid metabolizing enzymes/proteins regulate endogenous or exogenous lipid metabolism/cell steatosis^29^, we analyzed the impact of TNFAIP8 expression on lipid/fatty acid metabolizing enzyme expression (Supplementary Fig. 5c–e). The data demonstrated that the expression of TNFAIP8 in HCC cells increased that of FAS, SCD1, and SREBP1 mRNAs in HepG2 and SK-Hep1, and that of L-FABP1 and PPARG mRNAs in Hep3B cells (Supplementary Fig. 5c). Similarly, TNFAIP8 expression increased the expression of SCD1, ACC, PPAR-γ, and L-FABP1 proteins in HCC cells (Supplementary Fig. 5d). Conversely, TNFAIP8 knockdown reduced the expression of ACC and FASN, but no changes were observed in the expression of SCD1, PPARγ, L-FABP1, and SREBP1 proteins (Supplementary Fig. 5e). Collectively, the data presented in Fig. 6 suggest that TNFAIP8 binds with OA, modulates expression of lipid/fatty-acid metabolizing enzymes/proteins, and increases cell steatosis in HCC cells.

### TNFAIP8 regulates hepatic steatosis induced by EtOH, but not hepatic steatosis induced by a high-fat diet (HFD) in mice

Since autophagy plays an important role in lipid metabolism/cellular energy homeostasis^30^, we investigated whether there is an association between TNFAIP8, autophagy, and hepatic steatosis in mice fed with HFD or EtOH. Hematoxylin and eosin-stained liver sections clearly demonstrated that mice fed with HFD produced both macrovesicular (arrows) and microvesicular steatosis (arrowheads) in liver (Supplementary Fig. 6a, right panel), and such changes were absent in mice fed the chow diet (Supplementary Fig. 6a, left panel) suggesting that HFD induced hepatic steatosis in mice. On the other hand, no significant changes in expression of TNFAIP8 mRNA/protein and autophagy markers (Beclin-1 and LC3β I/II) were observed in liver tissues from HFD compared with chow diet-fed mice (Supplementary Fig. 6b–d) suggesting hepatic steatosis induced by HFD in mice is not associated with TNFAIP8.

**Fig. 7:**
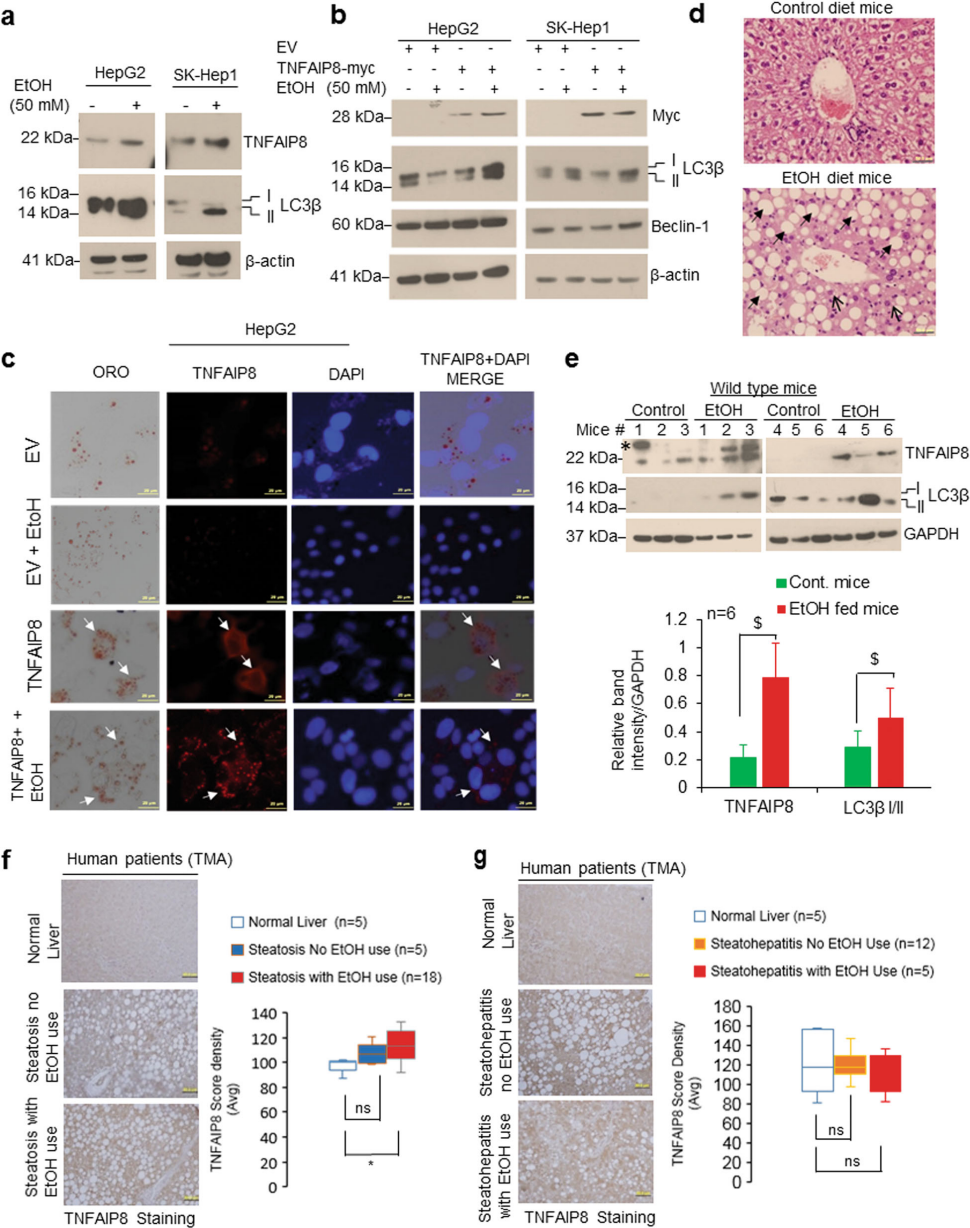
TNFAIP8-autophagy axis is associated with hepatic steatosis induced by EtOH. **a** HCC cells were treated with 50 mM EtOH for 30 h, and lysates were immunoblotted with indicated antibodies. **b** HCC cells were transfected with EV or TNFAIP8-Myc plasmid for 18 h and treated with 50 mM EtOH for 24 h and lysates were immunoblotted indicated antibodies. **c** HCC cells were grown on a coverslip and transfected with EV or TNFAIP8-Myc plasmid for 18 h and treated with 50 mM EtOH and 100 µM OA for 24 h. Cells were stained with Oil Red O and TNFAIP8-Myc protein as described in materials and methods section and observed under an Olympus BX60 fluorescent microscope with the bright field (×40 objective) and photographed. Arrow indicates TNFAIP8 expression and cell steatosis. **d** Representative hematoxylin and eosin (H&E, original magnification ×400) stained liver sections of male C57BL/6J mice pair-fed chronic ethanol (EtOH) or control diets for 8 weeks. **e** C57BL/6J mice pair-fed chronic ethanol (EtOH) or control diets for 8 weeks, mice were euthanized, and 50 μg of liver lysates were immunoblotted with indicated antibodies (upper panel). * = non-specific band. Indicated protein levels from the upper panel were quantified using ImageJ software (https://imagej.nih.gov/ij/) and plotted (lower panel). ^$^*P* < 0.05 relative to control mice. **f** TNFAIP8 expression is higher in hepatic steatosis human patients with a history of alcohol use: left and right panels: representative images and quantification of TNFAIP8 expression in liver tissue, including normal liver tissues, liver steatosis without alcohol use, and liver steatosis with a history of alcohol use. **g** Left panel: Representative images of TNFAIP8 expression in normal liver, steatohepatitis (NASH) with no history of alcohol use, and steatohepatitis with alcohol use. Right panel: the overall quantification of TNFAIP8 expression in normal liver tissue, liver steatosis without a history of alcohol use, and liver steatosis with a history of alcohol use. TNFAIP8 expression was analyzed and quantified from TMA. Boxplots show median and interquartile range with whiskers indicating the total range of TNFAIP8 expression. Data are expressed as the mean ± SEM  from 6 (**e**) mice or mean from 5–18 (**f**, **g**) human samples. *P < 0.05 relative to normal liver. ns: not significant.

Next, we analyzed the role of TNFAIP8 in EtOH-mediated cell steatosis in HCC cells and hepatic steatosis in mice fed chronic EtOH. In HCC cell lines, treatment with EtOH increased both endogenous TNFAIP8 and LC3β-II (Fig. 7a). TNFAIP8 overexpression and EtOH treatment induced LC3β I/II and Beclin-1 expression (Fig. 7b, left and right panels) and increased oleic acid-mediated cell steatosis in HepG2 cells (Fig. 7c). Further, we investigated the association between TNFAIP8/autophagy biomarker LC3B expression and EtOH-mediated hepatic steatosis in vivo using alcoholic C57BL/6J mouse model. We observed that chronic EtOH feeding produced both macrovesicular (indicated by arrows) and microvesicular steatosis (indicated by arrowheads) in mouse liver compared with mice fed with the control diet (Fig. 7d, upper and lower panels). Mice fed EtOH showed significantly increased expression of hepatic TNFAIP8 and induction of autophagy marker LC3B compared with control diet-fed mice (*n* = 6) (Fig. 7e, upper and lower panels).

We also analyzed the association of TNFAIP8 expression and hepatic steatosis in human patients with a history of EtOH use (*n* = 18) or no history of EtOH use (*n* = 5) (Fig. 7f). Immunohistochemical analysis and quantification of TNFAIP8 expression revealed that, compared with normal liver, TNFAIP8 expression was significantly higher in human hepatic steatotic patients with a history of alcohol use whereas the expression was not significantly associated with steatotic patients with no history of alcohol use (Fig. 7f, left and right panels). In contrast, TNFAIP8 expression was not significantly associated with patients with EtOH-mediated steatohepatitis (NASH) with or without a history of alcohol use or compared with the normal liver (Fig. 7g, left and right panel). Collectively, the data presented in Fig. 7 suggest that higher expression of TNFAIP8 and LC3B is associated with hepatic steatosis in mice induced by EtOH but not with HFD and higher expression of TNFAIP8 is associated with steatotic livers of human patients which have a history of EtOH use.

## Discussion

AFLD and NAFLD represent a spectrum of disease from steatosis to severe forms of injury, including NASH, cirrhosis, and HCC^31–33^. While a large number of associated molecular pathways have been described, the precise molecular events that promote the progression from steatosis to NASH to HCC are not defined^34,35^. In the current study, we identified a novel role of TNFAIP8 in early development and progression of liver diseases that underlie HCC progression. We investigated the molecular mechanism by which TNFAIP8 modulates early liver diseases or HCC progression using in vivo mouse models or in vitro using HCC cell models and liver cancer patient’s (TMA) samples. Our data suggest that TNFAIP8 enhances liver cancer cell survival and clonogenic potential, and increases resistance against anti-liver cancer drugs, sorafenib, and regorafenib by downregulation of cell apoptosis. These data are consistent with previous findings that TNFAIP8 is an oncogenic molecule known to induce chemotherapeutic drug resistance in various human cancers^4,36,37^.

Autophagy controls cell homeostasis by degradation and elimination of misfolded/aggregated proteins and damaged organelles through the lysosome^38,39^. In cancers, autophagy can modulate tumor suppression as well as tumor promotion^14,40,41^. Autophagy is involved in recovery from cellular stress induced by chemotherapeutic reagents, hence contributing to drug resistance^42,43^ and plays an important role in lipid metabolism, lipid homeostasis, and metabolic control^30,44^. TNFAIP8 has been shown to modulate autophagy in several model systems^12,15–17^. In an earlier study, Kristensen et al. demonstrated that autophagy-related protein 3 (ATG3) is an interacting partner of TNFAIP8^25^ and recently we confirmed TNFAIP8-ATG3 interaction in prostate cancer cells^12^. In the present study, we show that autophagy-related protein 7 (ATG7) is also as an interacting partner of TNFAIP8 suggesting that TNFAIP8 interacts with two key autophagosome proteins, ATG3 and ATG7. Since autophagy requires more than 30 ATG proteins to form an autophagosome, the regulation of each ATG protein is tightly regulated by several post-translational modifications including phosphorylation and oxidation of key catalytic thiol residues^26,45,46^. Crystal structures of yeast ATG3 and ATG7 suggest that each enzyme possesses a highly conserved and closely aligned catalytic site, which allows transfer of LC3^47^. Under physiological conditions, the interaction of ATG3 and ATG7 regulates LC3 transfer and lipidation of LC3. However, under oxidative stress conditions, rapid oxidation of catalytic thiols of ATG3 and ATG7 inhibits lipidation of LC3 and hence inhibits autophagy^26,46^. Our data indicate that, under physiological conditions, TNFAIP8 interacts with ATG3 and ATG7 and facilitates LC3 lipidation in HCC cells. Since TNFAIP8 is induced in response of TNFα, our data further suggest that exposure of TNFα (30 ng/ml) increases TNFAIP8 and promotes LC3 lipidation through interaction with ATG3 and ATG7 proteins.

**Fig. 8:**
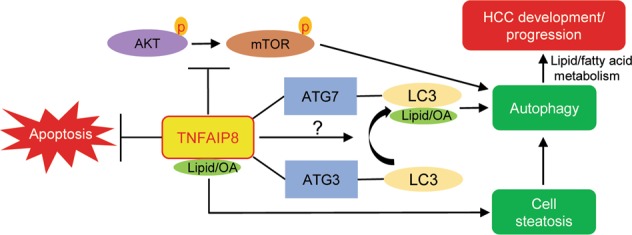
A molecular model represents the role of TNFAIP8 in regulation of ATK/mTOR inhibition, autophagy induction, cell steatosis, and HCC progression.

Recently, the crystal structure of mouse TNFAIP8 complex with phosphatidylethanolamine (PE), (a phospholipid involved in LC3 lipidation) was determined, and the data suggest that PE binds with TNFAIP8. Furthermore, the data revealed that under the influence of insulin, TNFAIP8 inhibits autophagy by the formation of TNFAIP8-PE-Gαi3 (Gαi3 subunit of the heterotrimeric G protein Gi3) ternary complex^16^. The study also indicates that substrates such as oleic acid can bind with TNFAIP8. We performed molecular docking and ELISA studies and demonstrated that fatty acids, including oleic acid, bind with TNFAIP8. On the other hand, recent reports also suggest that unsaturated fatty acid, including oleic acid induce non-canonical autophagy^48^, which occurs in the absence of some of the key autophagy-related proteins (ATG), through the unconventional biogenesis of canonical autophagosomes^49^. As depicted in Fig. 8, our combined data suggest a model whereby TNFAIP8 promotes autophagy through inhibition of AKT/mTOR, binding with autophagosome proteins, induction/stabilization of key proteins associated with autophagosome formation (Beclin-1, 4EBP1, ATG3, and ATG7), and lipidation of LC3 (Fig. 8). Furthermore, our data indicate that TNFAIP8 binds with OA and promotes cell steatosis as evidenced by increases in intracellular lipid droplet formation. This is consistent with our observations that TNFAIP8 binds with fatty acids and alters expression of fatty acid and lipid metabolizing enzymes. Our cumulative data, along with additional published data lead to a model where increased autophagy stress response acts to limit accumulation of lipid in lipid droplets in steatotic liver, but over time pathophysiological changes in autophagic function alter cellular lipid metabolism and promotes HCC development^50,51^.

The biological significance of TNFAIP8-mediated induction of autophagy, as well as the subsequent induction of cell steatosis was further examined in the obese mouse model and chronic EtOH fed mouse model. We demonstrated that higher expression of TNFAIP8 is associated with hepatic steatosis in mice fed EtOH. However, this association was not seen in steatotic mouse livers after HFD, suggesting that TNFAIP8 may modulate hepatic steatosis differently in NAFLD and AFLD. This is significant and surprising given that both fatty liver diseases initiate from simple hepatic steatosis and progress to NASH, cirrhosis, and HCC^52–54^. It is notable, though, that chronic alcohol consumption is ultimately linked to impaired autophagy, thus escalating the pathogenic progression to ALD^55^. Increased TNFAIP8 expression may be a cellular response to counteract this effect of alcohol. In agreement with above findings, we also observed that compared with normal liver, TNFAIP8 expression is significantly associated with hepatic steatotic human patients with a history of alcohol use but not with steatotic patients without alcohol use.

In conclusion, our data showed that higher expression of TNFAIP8 increased cell steatosis and autophagy in liver cancer cells. TNFAIP8 overexpression also inhibited apoptosis and increased HCC cell survival/drug resistance. We uncovered several mechanistic events that underpin these TNFAIP8-mediated phenotypic changes. Since these TNFAIP8-induced cellular events all have significance in early liver disease and progression to HCC, targeting TNFAIP8 activity in early liver diseases may provide therapeutic benefit for specific patient populations. Indeed, our data linked TNFAIP8 to steatotic events in alcohol mediated fatty liver disease development.

## Supplementary information

Legends for Suppl. material

Supplementary Table 1

Expression of TNFAIP8 isoforms in HCC cells

Effect of sorafenib and regorafenib on the expression of TNFAIP8 in HepG2 and SK-Hep1 cells

TNFAIP8 interacts with ATG7

ELISA

TNFAIP8 regulates cell steatosis by modulation of lipid/fatty-acid metabolizing enzyme expression

TNFAIP8 is not associated with hepatic steatosis induced by a high-fat diet in mice
