# Supplementary material for: TNFAIP8 regulates autophagy, cell steatosis, and promotes hepatocellular carcinoma cell proliferation
Source: Cell Death Dis. 2020 Mar 9;11(3):178. doi: 10.1038/s41419-020-2369-4 (PMC7062894; doi:10.1038/s41419-020-2369-4)
Supplement: Supplementary file 1 — Legends for Suppl. material [file 41419_2020_2369_MOESM1_ESM.docx]

**Supplementary Table 1 legend**

Set of primers used for the analysis of TNFAIP8, TNFAIP8 isoforms expression, and lipid, and fatty acid metabolic gene expression in RT/qPCR.

**Supplementary Figure legends**

**Supplementary Fig. 1 Expression of TNFAIP8 isoforms in HCC cells.** **a** & **b** Expression of TNFAIP8 mRNA and protein from indicated HCC cell lines were analyzed by RT/qPCR (n=3) and immunoblotting, respectively. **c** & **d** RT-PCR: Total RNA from HepG2, SK-Hep1, and Hep3B cells were isolated, and after cDNA synthesis, the expression of TNFAIP8 isoforms were amplified by PCR. The PCR products were electrophoresed on a 1.5% agarose gel stained with ethidium bromide. NC–negative control (no cDNA). The PCR-amplified bands were extracted from the agarose gel and sequenced to confirm the identity of the isoforms. Arrows indicate non-specific bands. SE-Short exposure. LE- Long exposure. WB- Western blotting.

**Supplementary Fig. 2 Effect of sorafenib and regorafenib on the expression of TNFAIP8 in HepG2 and SK-Hep1 cells.** **a** HepG2 and SK-Hep1 cells were grown in 6-well plates and treated with 2.5, and 5 µM of sorafenib or 0.5 and 1 µM regorafenib for 30h and cell lysates were immunoblotted with indicated antibodies. **b** Similarly, HepG2 and SK-Hep1 cells were grown in 6-well plates and treated with 2.5, and 5 µM of sorafenib or 0.5 and 1 µM regorafenib for 30h and the expression of TNFAIP8 mRNA was analyzed by RT/qPCR (n=3). Sora- Sorafenib. Rego-Regorafenib. **c** TNFAIP8 induced autophagy in SK-Hep1 cells. SK-Hep1 cells were grown on coverslips in 6-well plates and pre-treated with the autophagy inhibitor, 3-MA for 8h and transfected with EV or TNFAIP8-Myc plasmids as indicated. Cells were washed with PBS and then stained with Cyto-ID green fluorescence reagents and anti-Myc-TNFAIP8 rabbit antibody as described in materials and methods section. Cells were imaged using an Olympus BX60 fluorescent microscope (40X objective) and photographed. **d** EV and TNFAIP8-stable-expressing HepG2 cells were grown on coverslips in 6 well plates and treated with 3-MA for 24h. Cells were stained with LC3β I/II-related Cyto-ID green fluorescence reagents at 37°C for 1h. Cells were fixed, stained with Vectashield medium containing nuclear DAPI stain, observed under an OlympusBX60 fluorescent microscope (20X objective), and photographed. Arrow indicates LB3B related puncta formation.

**Supplementary Fig. 3** **TNFAIP8 interacts with ATG7.** **a** Two milligrams of PC3 cell lysates were IPed with control IgG or TNFAIP8 antibody. Immunocomplexes were resolved by SDS-PAGE and gel stained with simplyblue (left panel), and TNFAIP8 interacting proteins were identified by Mass Spectrometry. TNFAIP8 and ATG7 unique peptides identified from Mass Spectrometry data are presented (right panel). **b** Interaction of TNFAIP8 with ATG7 was analyzed after TNFAIP8 immunoprecipitation followed by Mass Spectrometry. Mass Spectrometry identified TNFAIP8, and ATG7 peptides were analyzed by using Scaffold software (<http://www.proteomesoftware.com/products/scaffold/>) and presented. One of the MS ionization spectrum of TNFAIP8 and ATG7 peptides were presented.

**Supplementary Fig. 4 ELISA.** **a** The binding pattern with stearic acid, myristic acid, linoleic acid, and cholesterol with hTNFAIP8His-tagged protein was determined by ELISA. SA-Stearic acid. MA- myristic acid. LNA-linoleic acid. Chol- Cholesterol. **b** Effect of *cis* and *trans* oleic on liver cancer cell steatosis. HepG2, SK-Hep1, and Hep3B cells were treated with 100 (µM) *cis* and *trans* oleic acid, separately. Cells were fixed, stained with Oil Red O stain, and mounted using Permount solution. Slides were observed under a light microscope using 40X objective and photographed. **c** HCC cells were treated with 25µM and 50µM of *cis* and *trans* oleic as indicated for 30h, and cell lysates (50 µg) were immunoblotted with indicated antibodies.

**Supplementary Fig. 5 TNFAIP8 regulates cell steatosis by modulation of lipid/fatty acid metabolizing enzyme expression.** TNFAIP8 knockdown reduced cell steatosis in HepG2, SK-Hep1, and Hep3B cells. **a** HepG2, SK-Hep1, and Hep3B cells were grown on coverslips in 6-well plates and transfected with control siRNA or TNFAIP8 siRNA for 24h and treated with 100 µM oleic acid for 24h. After Oil Red O staining, slides were observed under a light microscope using 40X objective and photographed. **b** The number of ORO-stained lipid droplets present in the control siRNA transfected and TNFAIP8 siRNA transfected cells were measured and plotted. Data represent mean ± SEM from 3 experiments. **P<0.1* compared to control siRNA transfected and OA treated HepG2 and Hep3B cells. ****P<0.001* compared control siRNA transfected and OA treated SK-Hep1 cells. **c** HCC cells were transfected with EV or TNFAIP8-Myc plasmid for 30h, and the effect of TNFAIP8 expression on lipid/fatty acid metabolizing enzymes mRNA expression were analyzed by RT/qPCR (n=3). **d** & **e** HCC cells were transfected with EV, and TNFAIP8-Myc plasmids (d) or control siRNA or TNFAIP8 siRNA (e) for 30h and lysates were WB with indicated antibodies. ααα*P*<0.001, αα*P*<0.01, ιιι *P*<0.001, β *P*<0.05, βββ *p<0.001* relative to EV transfected cells.

**Supplementary Fig. 6** **TNFAIP8 is not associated with hepatic steatosis induced by a high-fat diet in mice. a** Representative hematoxylin and eosin (H & E, original magnification ×400) stained liver sections of male C57BL/6J mice fed with a chow diet (Control diet, 12% calories as fat) or a high-fat diet (HFD, 45% calories as fat) for 16 weeks. **b** Total RNAs were isolated from mice livers (n=5) fed with control-diet or HFD, and after cDNA synthesis, the effect of HFD on *TNFAIP8* gene expression in liver tissues was analyzed by RT/qPCR. **c** C57BL/6J mice fed with a chow diet or HFD for 16 weeks. Mice were euthanized, and livers were isolated, lysed, and fifty micrograms lysates were immunoblotted with indicated antibodies. **d** Indicated protein levels from (c) were quantified using ImageJ software (<https://imagej.nih.gov/ij/>). Data represent mean ± SEM from 5 (b) or 4 (c & e) animals each group. NS- not significant.
