# Supplementary material for: TNFAIP8 regulates autophagy, cell steatosis, and promotes hepatocellular carcinoma cell proliferation
Source: Cell Death Dis. 2020 Mar 9;11(3):178. doi: 10.1038/s41419-020-2369-4 (PMC7062894; doi:10.1038/s41419-020-2369-4)
Supplement: Supplementary file 2 — Supplementary Table 1 [file 41419_2020_2369_MOESM2_ESM.docx]

**Supplementary Table 1**

| TNFAPI8 FP: 5’- CTTTGACCGGAATGTGTTATCCA-3’  TNFAPI8 FP: 5’ – CAAGGCAGCCAAAAATTCACAA-3’  FAS FP: 5’-CTAGGTTTGATGCCTCCTTCTT-3’  FAS RP: 5’-GATGGCTTCATAGGTGACTTCC-3’  PPARγ FP: 5’-CTCAAACGAGAGTCAGCCTTTA-3’  PPARγ RP: 5’-GTGGGAGTGGTCTTCCATTAC-3’  SCD1 FP: 5’-CTCTTTCTGCTCTGCCATCTT-3’  SCD1 RP: 5’-CCCGACTTCACCTCCTTAAATC-3’  L-FABP FP: 5’-GGAATGTGAGCTGGAGACAA-3’  L-FABP RP: 5’-AGTTCGGTCACAGACTTGATG  SREBP1 FP: 5’-GAGCCATGGATTGCACTTTC-3’  SREBP1 RP: 5’-AGCATAGGGTGGGTCAAATAG-3’  ACC FP: 5’-GCAGGTCACACGTCTCTTTAT-3’  ACC RP: 5’-CCAGCCTGTCATCCTCAATATC-3’  IL6 FP: 5’-GTAGTGAGGAACAAGCCAGAG-3’  IL6 rp: 5’-GGACTGCAGGAACTCCTTAAA-3’  GAPDH FP: 5’- CCACCCAGAAGACTGTGGAT -3’  GAPDH RP: 5’- GTTGAAGTCAGAGGAGACCACC -3’ |
| --- |
| For TNFAIP8 Isoforms expression  Isoform 1 FP: 5ʹ-CGAGTACATGTGAGCGGTAAT-3ʹ  Isoform 1 RP: 5ʹ-GATCTTCTCTGCCTCCTTCTTG-3ʹ  Isoform 2 FP: 5ʹ-AC CGAGAGAGCAGAGAACT-3ʹ  Isoform 2 RP: 5ʹ-GATCTTCTCTGCCTCCTTCTTG-3ʹ  Isoform 3 FP: 5ʹ-GGCTGT CCGGCTTCTTTAT-3ʹ,  Isoform 3 RP: 5ʹ-GATCTTCTCTGCCTCCTTCTTG-3ʹ  Isoform 4 FP: 5ʹ- AGTCCATCCC TGTTGTGAATG-3ʹ  Isoform 4 RP: 5ʹ-GATCTTCTCTGCCTCCTTCTTG-3ʹ  Isoform 5 FP: 5ʹ-AAGTGCAGTGG TGAGATCATAG-3ʹ  Isoform 5 RP: 5ʹ-GATCTTCTCTGCCTCCTTCTTG-3ʹ |
