# Supplementary figures and images for: TNFAIP8 regulates autophagy, cell steatosis, and promotes hepatocellular carcinoma cell proliferation
Source: Cell Death Dis. 2020 Mar 9;11(3):178. doi: 10.1038/s41419-020-2369-4 (PMC7062894; doi:10.1038/s41419-020-2369-4)

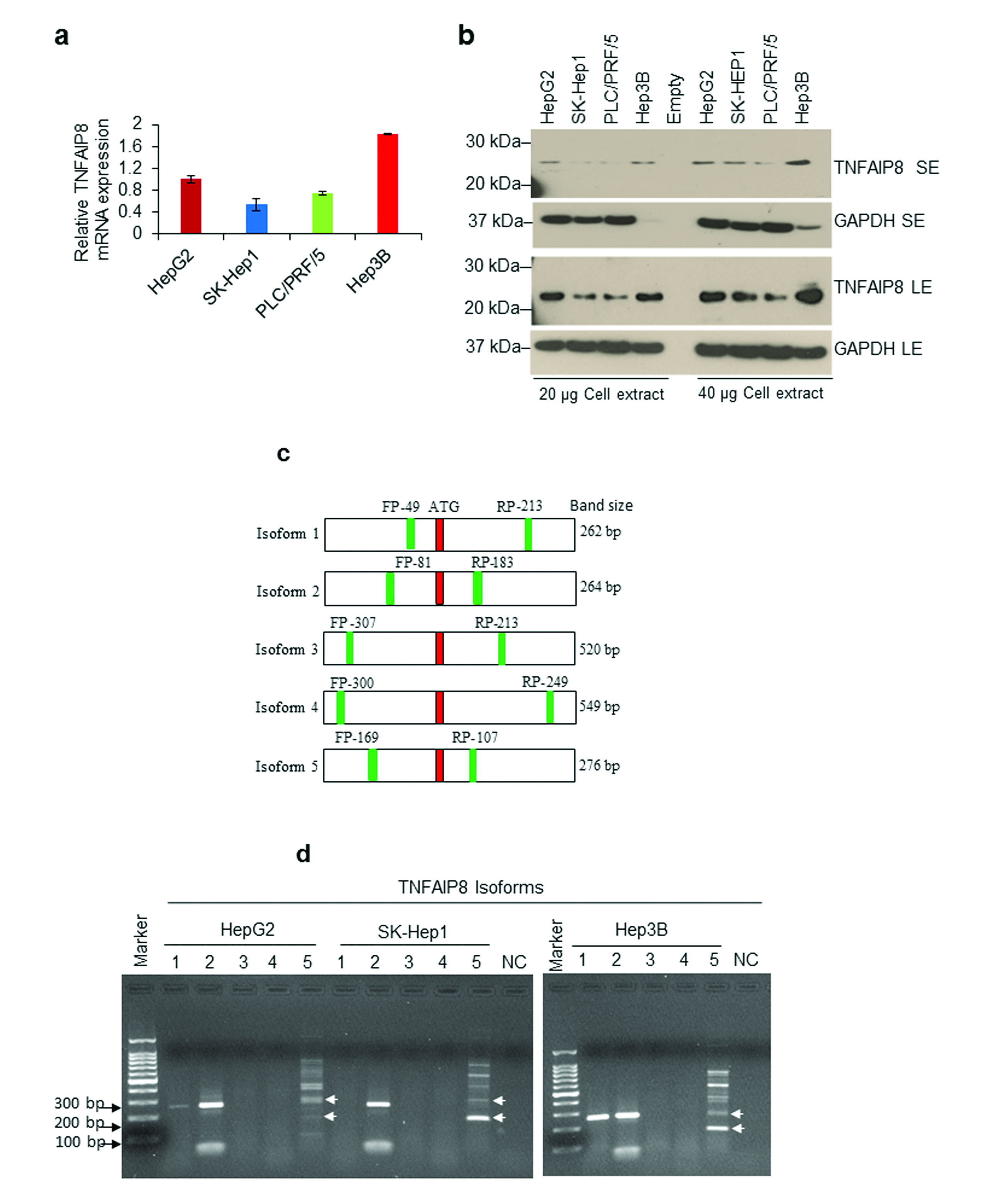

Supplement: Supplementary file 3 — Expression of TNFAIP8 isoforms in HCC cells [file 41419_2020_2369_MOESM3_ESM.tif]

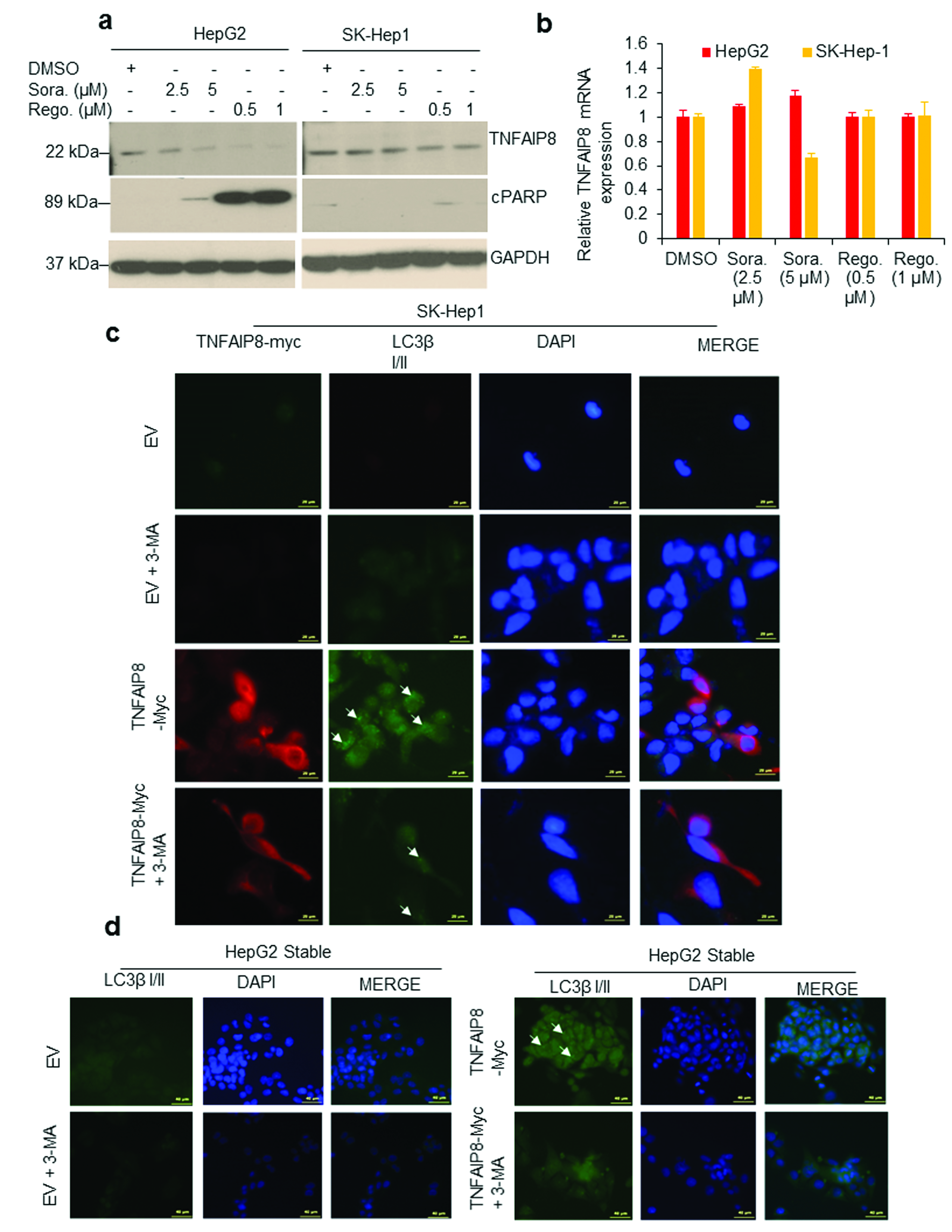

Supplement: Supplementary file 4 — Effect of sorafenib and regorafenib on the expression of TNFAIP8 in HepG2 and SK-Hep1 cells [file 41419_2020_2369_MOESM4_ESM.tif]

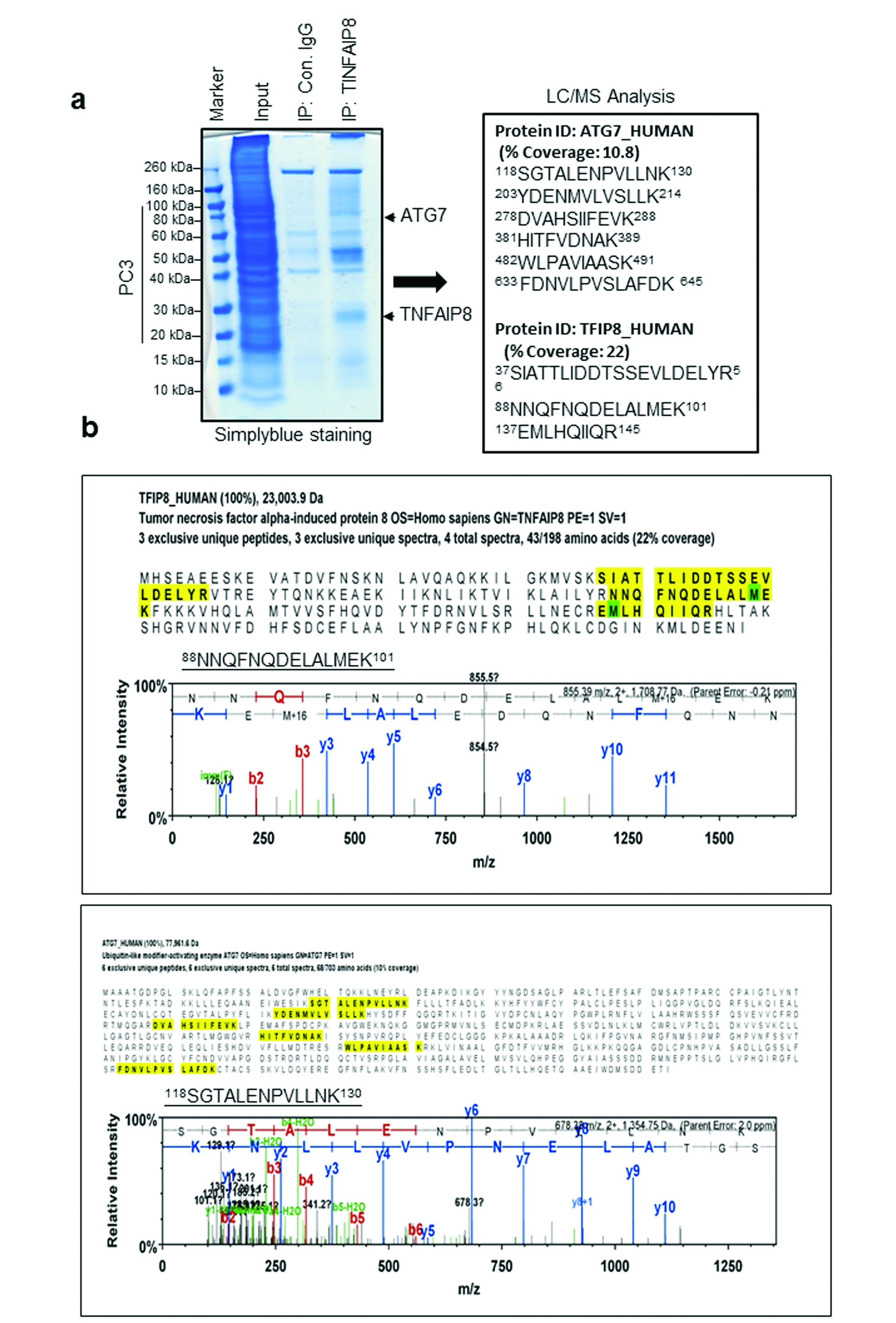

Supplement: Supplementary file 5 — TNFAIP8 interacts with ATG7 [file 41419_2020_2369_MOESM5_ESM.tif]

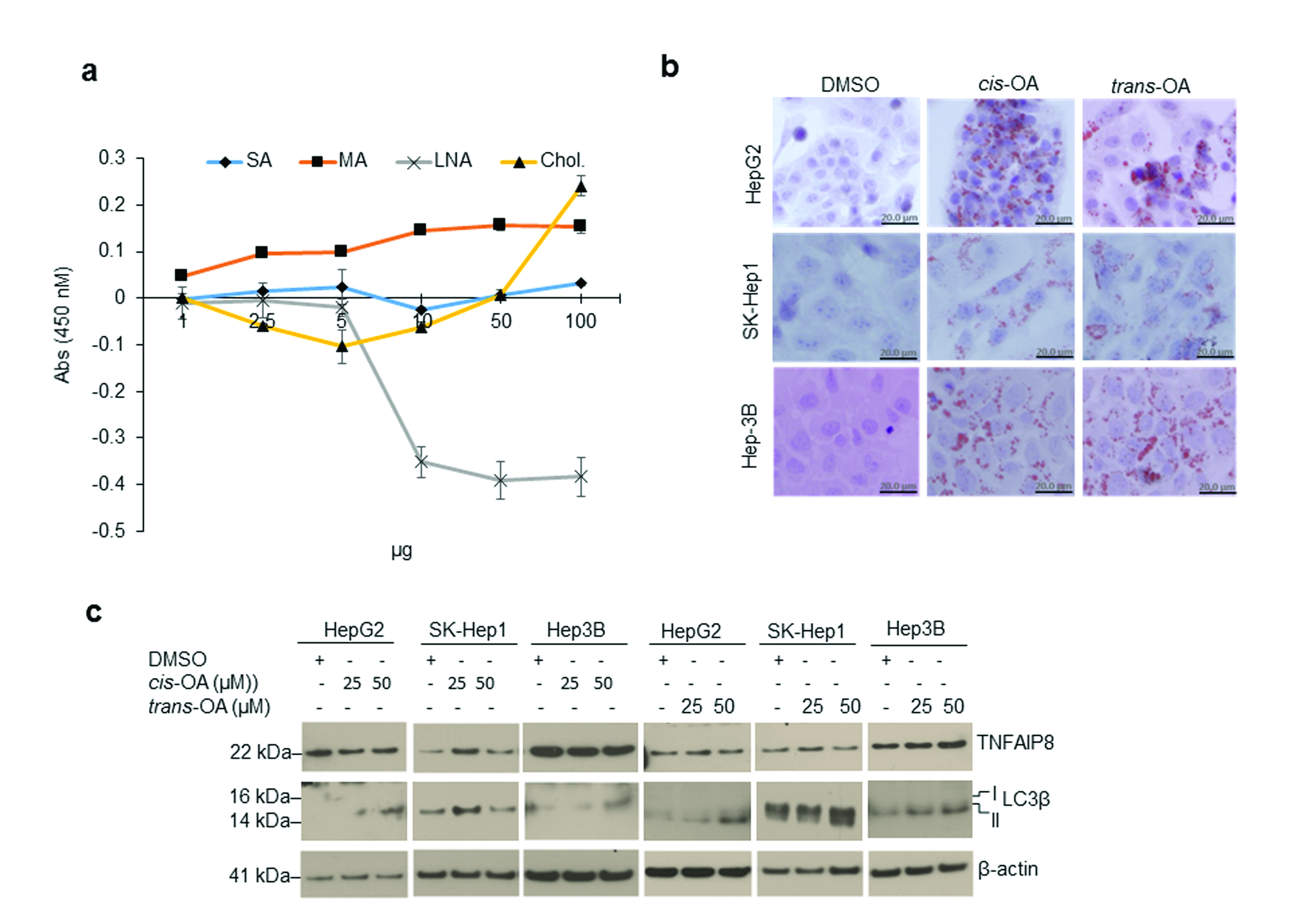

Supplement: Supplementary file 6 — ELISA [file 41419_2020_2369_MOESM6_ESM.tif]

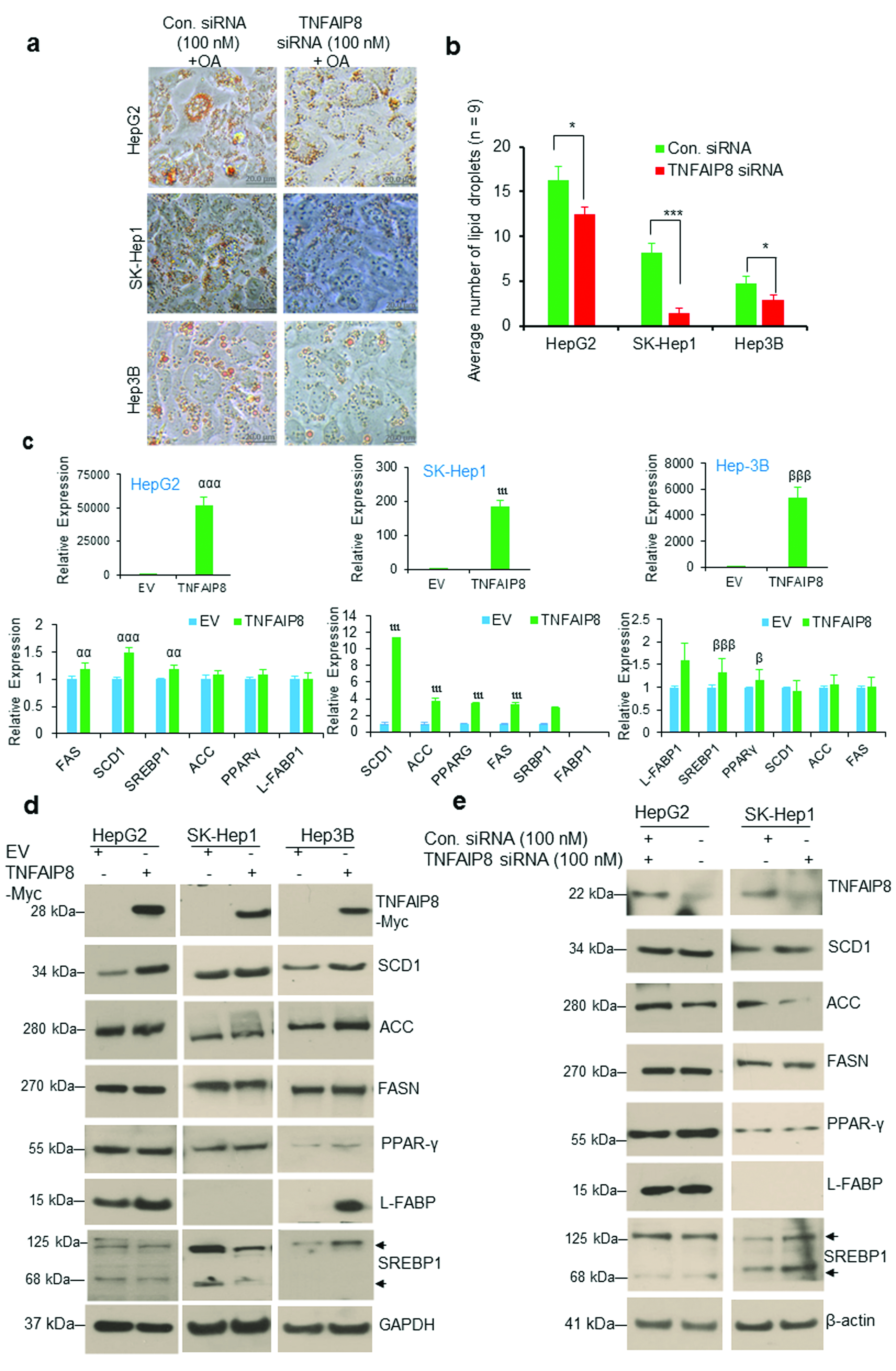

Supplement: Supplementary file 7 — TNFAIP8 regulates cell steatosis by modulation of lipid/fatty-acid metabolizing enzyme expression [file 41419_2020_2369_MOESM7_ESM.tif]

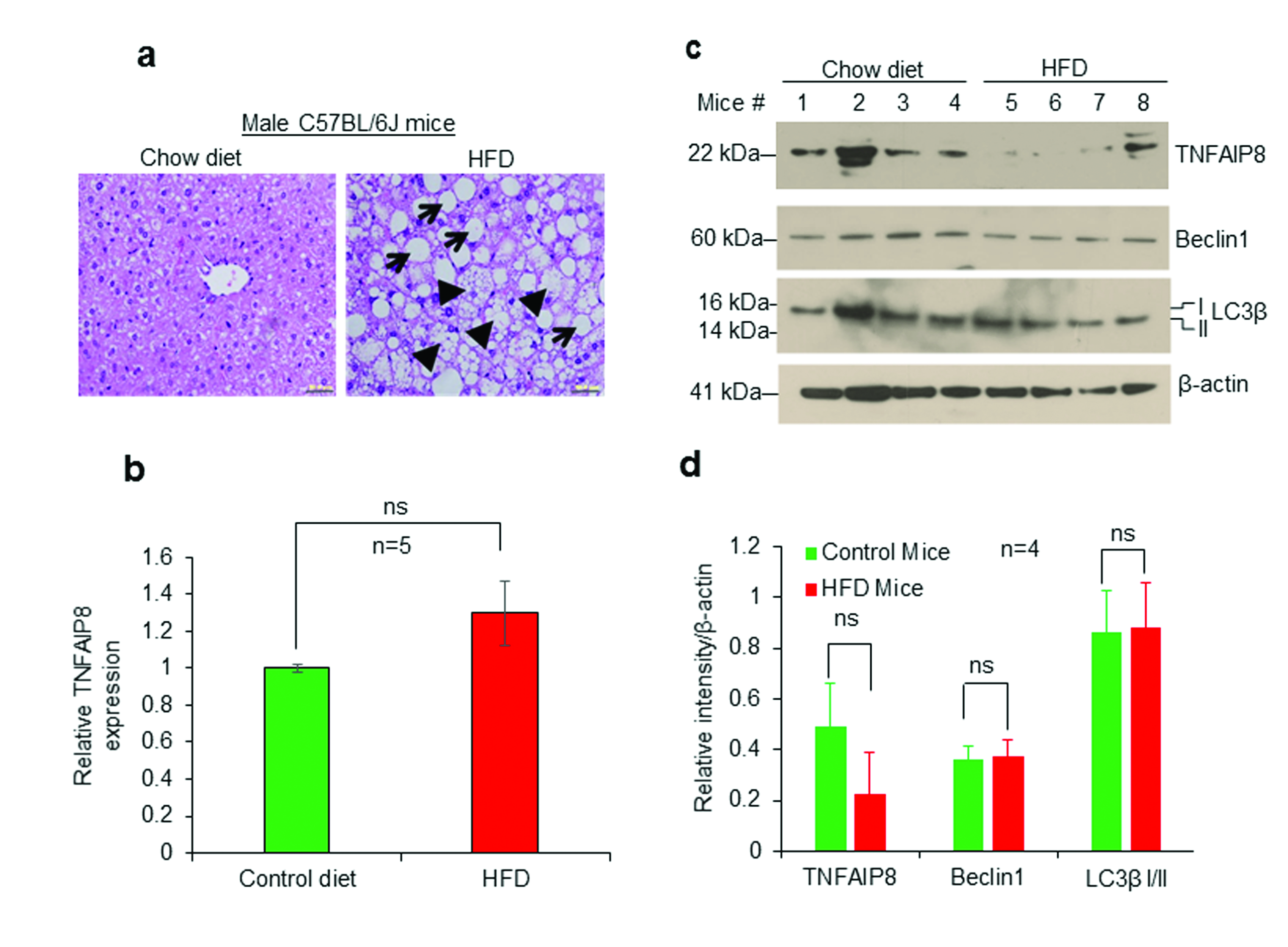

Supplement: Supplementary file 8 — TNFAIP8 is not associated with hepatic steatosis induced by a high-fat diet in mice [file 41419_2020_2369_MOESM8_ESM.tif]
